# Enhanced Solubility and Bioavailability of Clotrimazole in Aqueous Solutions with Hydrophobized Hyperbranched Polyglycidol for Improved Antifungal Activity

**DOI:** 10.1021/acsami.3c19388

**Published:** 2024-04-05

**Authors:** Daria Jaworska-Krych, Monika Gosecka, Mateusz Gosecki, Malgorzata Urbaniak, Katarzyna Dzitko, Anita Ciesielska, Ewelina Wielgus, Slawomir Kadlubowski, Marcin Kozanecki

**Affiliations:** † Centre of Molecular and Macromolecular Studies, Polish Academy of Sciences, Sienkiewicza 112, 90-363 Lodz, Poland; ‡ Department of Molecular Microbiology, Faculty of Biology and Environmental Protection, University of Lodz, Banacha 12/16, 90-237 Lodz, Poland; § Institute of Applied Radiation Chemistry, Lodz University of Technology, Wroblewskiego 15, 93-590 Lodz, Poland; ∥ Department of Molecular Physics, Faculty of Chemistry, Lodz University of Technology, Zeromskiego 116, 90-924 Lodz, Poland

**Keywords:** hydrophobic drug, clotrimazole, drug carrier, structured fluid, Bingham fluid, drug permeability, intravaginal antifungal therapy

## Abstract

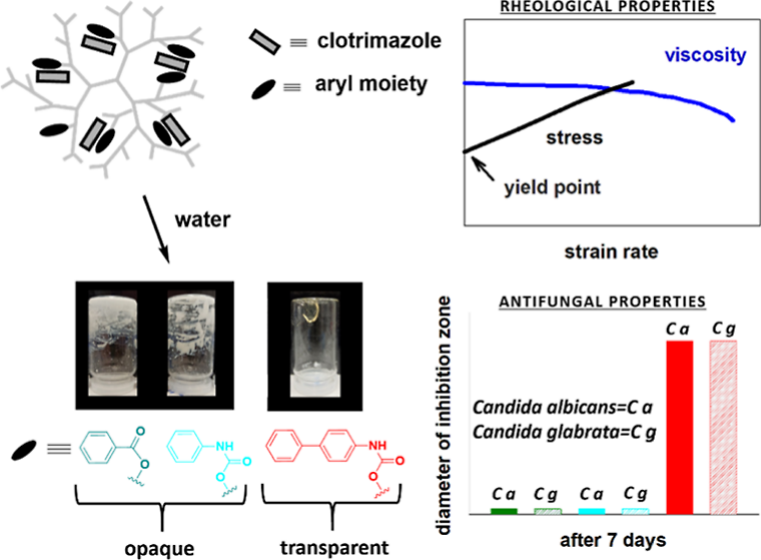

The poor solubility of clotrimazole in the aqueous medium and the uncontrolled removal of the drug-loaded suppository content limit its effectiveness in the treatment of vulvovaginal candidiasis. We present here the aqueous formulations of clotrimazole in the form of non-Newtonian structured fluids, *i.e.*, Bingham plastic or pseudoplastic fluids constructed of hyperbranched polyglycidol, HbPGL, with a hydrophobized core with aryl groups such as phenyl or biphenyl. The amphiphilic constructs were obtained by the modification of linear units containing monohydroxyl groups with benzoyl chloride, phenyl isocyanate, and biphenyl isocyanate, while the terminal 1,2-diol groups in the shell were protected during the modification step, followed by their deprotection. The encapsulation of clotrimazole within internally hydrophobized HbPGLs using a solvent evaporation method followed by water addition resulted in structured fluids formation. Detailed Fourier-transform infrared spectroscopy (FTIR) and differential scanning calorimetry (DSC) analyses performed for aryl-HbPGLs with clotrimazole revealed the difference in drug compatibility among polymers. Clotrimazole in biphenyl-enriched HbPGL, unlike phenyl derivatives, was molecularly distributed in both the dry and the hydrated states, resulting in transparent formulations. The shear-thinning properties of the obtained fluid formulations make them injectable and thus suitable for the intravaginal application. Permeability tests performed with the usage of the Franz diffusion cell showed a 5-fold increase in the permeability constant of clotrimazole compared to drugs loaded in a commercially available disposable tablet and a 50-fold increase of permeability in comparison to the aqueous suspension of clotrimazole. Furthermore, the biphenyl-modified HbPGL-based drug liquid showed enhanced antifungal activity against both *Candida albicans* and *Candida glabrata* that was retained for up to 7 days, in contrast to the phenyl-HbPGL derivatives and the tablet. With their simple formulation, convenient clotrimazole/biphenyl-HbPGL formulation strategy, rheological properties, and enhanced antifungal properties, these systems are potential antifungal therapeutics for gynecological applications. This study points in the synthetic direction of improving the solubility of poorly water-soluble aryl-enriched pharmaceuticals.

## 1 Introduction

Vulvovaginal candidiasis, a common fungal inflammatory disease caused mainly by *Candida albicans* (in about 90% of cases), with most of the remaining cases caused by *Candida glabrata*, is responsible for a third of all cases of vulvovaginitis in reproductive-aged women.^1,2^ Although *C. glabrata* is less prevalent, it is a substantial threat, particularly in immune-compromised individuals, *i.e.*, patients undergoing chemotherapy or antimetabolic drugs, HIV-infected patients, or transplant patients.^3−5^ Other major factors contributing to acute vulvovaginal candidiasis include estrogen use, elevated endogenous estrogen levels (due to pregnancy or obesity),^6^ diabetes, and the use of broad-spectrum antibiotics.^3−6^

In the therapy of vulvovaginal candidiasis, clotrimazole (CLT), *i.e.*, an imidazole derivative belonging to the azole class of antifungal compounds, is commonly used in the form of creams, ointments, globules, and tablets.^7^ It shows mainly the activity against *C. albicans*, along with very low anti-*C. glabrata* activity.^5,8^ The bioavailability of CLT is limited in the aqueous medium due to its low solubility in water (0.49 μg/mL)^9^ and, in consequence, therapeutic efficiency is reduced. Furthermore, in the case of intravaginal therapy, frequent administration of globules or tablets is required as the content of the therapeutic formulation is often released uncontrollably.^7,10^ In fact, the affected site is often not adequately penetrated by the bioactive compound, its absorbed amount is below the therapeutic dose, leading to recurrent candidiasis and ultimately may be the cause of infertility.^11^ For this reason, the number of azole-resistant fungal infections is still increasing.^12−14^

The current challenge in intravaginal therapies is the formation of an efficient carrier of CLT, which can enhance its solubility in aqueous medium, prolonged drug interactions with the afflicted area, and provide suitable permeability of the vaginal mucosa with drug molecules. Additionally, it is expected to reduce the number of administrations of the therapeutic formulation required for recovery.

Prolonged drug interaction with the vagina can be attained by the use of hydrogel carriers. Their usage in the transport of CLT is, however, problematic as a result of the discrepancy between the hydrophilic nature of hydrogel systems and the hydrophobic character of the bioactive compounds. However, it can be overcome by the construction of hydrogels with the incorporation of distinct hydrophobized domains.^15^ Until now, the solubilization of azole-based antifungal drugs such as voriconazole in the aqueous medium was attained by embedding solid drug-loaded polymer particles in the hydrogel matrix.^16,17^ In the case of fluconazole, the suspension of drug particles coated with poly(ethylene glycol) was combined with the gelling mixture.^18^ The solubility of CLT in the aqueous medium was attained by the usage of water-miscible cosolvents or drug loading in the form of microemulsions or nanocapsules^19^ suspended into the aqueous solution of gelling polymers. Such a strategy of incorporation of poorly water-soluble drugs often requires, however, the usage of surfactants.^20^ The complete release of CLT from these hydrogel carriers was limited to several hours. Recently, a novel synthetic strategy of CLT-enriched hydrogels constructed of dynamic cross-links has been elaborated based on the usage of drug-loaded unimolecular micelles displaying prolonged CLT release reaching 40 h.^21−23^ This approach required, however, the use of a cross-linking agent for hydrogel formation.

In this work, we demonstrate the aqueous non-Newtonian structured fluids loaded with CLT constructed of a one-polymer component, *i.e.*, aryl-enriched polyether unimolecular micelles, without the need of adding any surfactant or additional cross-linking agent. Formulations of such simple construction based on internally hydrophobized hyperbranched polyglycidol, *h*HbPGL, with phenyl and biphenyl moieties exhibiting affinity to CLT ensure extended retention of the bioactive compound in the affected area and enhanced CLT bioavailability. The characteristics of CLT-loaded structured fluids make them promising carriers of hydrophobic drugs for efficient intravaginal therapy. The biocompatible character of HbPGL with a hydrophobized interior makes these formulations highly prospective as drug delivery systems.^22^

In this study, we present the relationship between the structural characteristics of the constructed unimolecular micelles, *i.e.*, the size of the aryl group, the type of linkage used for immobilization of the aryl group, the rheological properties of the prepared formulations, the solubilization ability of CLT and its permeability through the skin barrier, and antifungal properties against both *C. albicans* and *C. glabrata*. It was shown that the structured liquids of HbPGLs enriched with an aryl group with CLT could show better activity against *C. glabrata* compared with the free drug. The antifungal properties of the structured liquids were closely dependent on the size of the aryl group used to hydrophobize HbPGL, affecting the solubility of CLT in hyperbranched polymeric structures and the skin permeability. This work contributes to a better understanding of the material construction strategy and its biological function. Moreover, this work demonstrates the potential of structured fluids to be key drug carriers in gynecology in addition to hydrogel formulations.

## 2 Materials and Methods

### 2.1 Materials

1,1,1-Tris(hydroxymethyl)propane was purchased from Sigma-Aldrich. It was dissolved under reflux in acetone, precipitated with ethyl ether, and then dried prior to use. NaH (60 wt %) in mineral oil was purchased from Merck. The oil was removed by washing with dry 1,4-dioxane and then drying under reduced pressure. Glycidol (Sigma-Aldrich) was dried over CaH_2_ and distilled before use. Tetrahydrofuran (THF) was purchased from Sigma-Aldrich and dried over Na/K alloy. 2,2-Dimethoxypropane (TCI), benzoyl chloride, phenyl isocyanate (Alfa Aesar), and 4-biphenylyl isocyanate (Sigma-Aldrich) were used as received. *p*-Toluenesulfonic acid (PTSA) was purchased from Sigma-Aldrich and dried with benzene before use. CLT (Sigma-Aldrich) was used as received. Clodital MAX 500 mg, USP Health, a CLT-loaded commercially available tablet, was used as a reference.

### 2.2 Synthesis of HbPGL

**Scheme 1:**
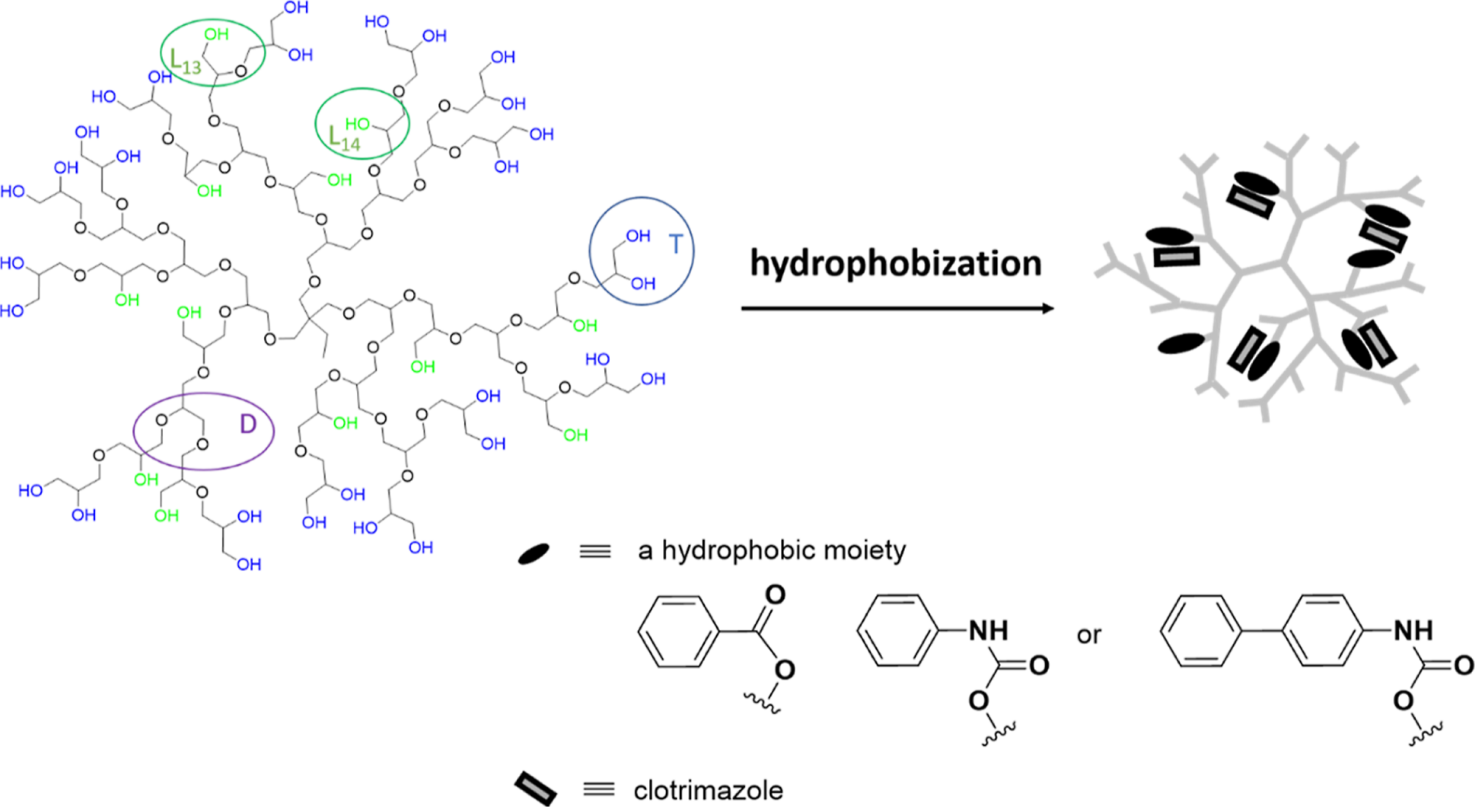
Schematic Representation of Internally Aryl-Enriched Hyperbranched Polyglycidol Applied for the Encapsulation of Clotrimazole, where **D** = Dendritic, **T** = Terminal, and **L**_**13**_ and **L**_**14**_ = Linear Constitutional Units

The synthesis of HbPGL was carried out in a thermostated glass reactor equipped with a steel mechanical stirrer under an argon atmosphere. Twenty percent of the hydroxyl groups of 1,1,1-tris(hydroxymethyl)propane (105 mg; 7.8 × 10^–4^ mol) were converted to alcoholates in THF using NaH (4.6 × 10^–4^ mol). Thirty five mL of glycidol was dropped into the reactor at a rate of 2 mL/h, and the polymerization was conducted for 24 h at 95 °C. The product was dissolved in methanol, twice precipitated into acetone, and dried. The polymer was then dissolved in deionized water and dialyzed using dialysis tubes MCWO = 3.5k. The structure of the synthesized polymer was characterized with ^1^H and ^13^C INVGATE NMR spectroscopy. The degree of branching of the synthesized neat HbPGL was 0.55. The molar fraction of dendritic (**D**) and total linear constitutional units **L**_**13**_ and **L**_**14**_ bearing monohydroxyl groups was 0.25 and 0.40, respectively, whereas the molar fraction of terminal units (**T**) containing diol moieties was 0.35. **D**, **L**_**13**_, **L**_**14**_, and **T** are denoted in Scheme 1. The weight-average molecular mass *M*_w_ was determined based on GPC results using water as an eluent and is equal to 12,000 g/mol, while the dispersity *D̵* = 1.8.

### 2.3 Hydrophobization of HbPGL Core with Phenyl and Biphenyl Urethane Moieties

HbPGL (20 g) of a molecular weight equal to 12,000 g/mol was chemically modified by the protection of 1,2-diol groups in reaction with 2,2-dimethoxypropane (96 mL, 0.78 mol) in the presence of PTSA (0.192 g, 1.11 mmol) by ultrasonication at 40 °C for 3 h. The crude product was diluted with chloroform and extracted three times with a saturated Na_2_CO_3_ solution to remove PTSA. The organic phase was dried over MgSO_4_ and dialyzed in chloroform for 24 h. The product was then dried under high vacuum conditions and analyzed by ^1^H and ^13^C INVGATE NMR spectroscopy in deuterated DMSO to confirm the complete conversion of terminal 1,2-diol groups to acetals (Acet), Figures S4–S6.

^1^H NMR (400 MHz, DMSO): δ (ppm) 4.84–4.36 (OH-L_13_, L_14_), 4.14 m, 1H, C*H*-PG (T Acet), 3.97 (m, 1H, C*H*(H)-PG (T Acet)), 3.80–3.20 (5H-HbPGL backbone, m, 1H, CH(*H*)-PG (T Acet)), 1.30 (3H-CH_3_ T Acetal), 1.26 (3H-CH_3_ T Acetal).

^13^C INVGATE NMR (400 MHz, DMSO): δ (ppm) 108.84 (C-T Acetal), 80.39 (CH-L_1,3_), 78.38 (CH-D), 74.69 (CH-T Acetal), 73.33 (CH_2_-2L_1,4_), 72.64–70.61 (CH_2_-2D,T), 69.91 (CH_2_-L_13_), 69.52–68.77 (CH-L_1,4_), 66.46 (CH_2_-T Acetal), 61.27 (CH_2_-L_1,3_), 27.09 (CH_3_-T Acetal), 25.85 (CH_3_-T Acetal).

HbPGL with protected 1,2-diol groups (3.5 g) in terminal units was dried with benzene, dissolved in pyridine (20 mL) under argon conditions, and heated to 50 °C. Then, a 4-biphenyl isocyanate solution in pyridine (0.106 g/mL) or 0.89 mL of phenyl isocyanate, respectively, was dropped. The reaction was carried out for 24 h. The reaction mixture was dialyzed against DMSO. After evaporation of the solvent, the degree of hydrophobization of all monohydroxyl units was determined on the basis of the ^1^H NMR spectrum recorded in deuterated DMSO-*d*_6_, comparing the integration of methyl protons from acetal groups in terminal units (1.15 and 1.35 ppm) and aromatic protons from biphenyl groups (7.10 to 7.75 ppm). In the case of the phenyl-HbPGL derivative, 66 mol % of all L_13_ and L_14_ units were hydrophobized, *i.e.*, 41 hydrophobic units per macromolecule (Figure S7), while for the biphenyl-enriched HbPGL derivative, 40 mol % of all L_13_ and L_14_ units were hydrophobized, *i.e.*, 24 hydrophobic units per macromolecule (Figure S9).

^1^H NMR (400 MHz, DMSO-*d*_6_): δ (ppm) for the BPh-HbPGL derivative 9.97–9.68 (1H-NH), 7.57 (6H-biphenyl), 7.40 (2H-biphenyl), 7.30 (1H-biphenyl), 5.01 (1H-CH-L_1,4-hydrophobic_), 4.89–4.44 (1H-OH-L_1,3_, L_1,4_), 4.24 (1H-CH_2_-L_1,3-hydrophobic_), 4.12 (1H-CH_2_-T_Acetal_), 3.94 (1H-CH_2_-T_Acetal_), 3.82–3.02 (HbPGl-backbone), 1.29 (3H-CH_3_ T_Acetal_), 1.23 (3H-CH_3_ T_Acetal_).

^1^H NMR (400 MHz, DMSO-*d*_6_): δ (ppm) for the Ph-HbPGL derivative 9.78–9.55 (1H-NH), 7.46-2H, 7.25-2H, 6.97-1H-phenyl, 4.97 (1H-CH-L_1,4-hydrophobic_), 4.86–4.38 (1H-OH-L_1,3_, L_1,4_), 4.20 (1H-CH_2_-L_1,3-hydrophobic_), 4.11 (1H-CH_2_-T_Acetal_), 3.94 (1H-CH_2_-T_Acetal_), 3.82–3.02 (HbPGl-backbone), 1.29 (3H-CH_3_ T_Acetal_), 1.23 (3H-CH_3_ T_Acetal_).

Subsequently, 1,2-diol groups of the polymer in terminal units were deprotected by the addition of an aqueous solution of 0.1 M HCl to the polymer solution in DMSO and stirred overnight at room temperature. The mixture was dialyzed against deionized water for 24 h, changing the solvent until it reached a neutral pH. The product was lyophilized and characterized by using ^1^H NMR spectroscopy in deuterated DMSO (Figures S8 and S10).

^1^H NMR (400 MHz, DMSO-*d*_6_): δ (ppm) for BPh-HbPGL derivative 9.98–9.65 (1H-NH), 7.57 (6H-biphenyl), 7.40 (2H-biphenyl), 7.30 (1H-biphenyl), 5.01 (1H-CH-L_1,4-hydrophobic_), 4.90–4.34 (1H-OH-L_1,3_, L_1,4_, T), 4.23 (1H-CH_2_-L_1,3-hydrophobic_), 4.11 (1H-CH_2_-L_1,3-hydrophobic_), 3.98–3.00 (HbPGL-backbone).

^1^H NMR (400 MHz, DMSO-*d*_6_): δ (ppm) for Ph-HbPGL derivative 9.80–9.49 (1H-NH), 7.45-2H, 7.25-2H, 6.97-1H-phenyl, 4.98 (1H-CH-L_1,4-hydrophobic_), 4.86–4.36 (1H-OH-L_1,3_, L_1,4_, T), 4.21 (1H-CH_2_-L_1,3-hydrophobic_), 4.09 (1H-CH_2_-L_1,3-hydrophobic_), 3.90–3.00 (HbPGL-backbone).

### 2.4 Hydrophobization of AC-HbPGL Core with Benzoate Groups (Ester Derivative)

HbPGL with protected 1,2-diol groups (3.5 g) in terminal units was dried with benzene, dissolved in pyridine (20 mL) under argon conditions, and cooled in an ice bath to 0 °C. Then, 0.92 mL of benzoyl chloride was dropped. The reaction was carried out for 24 h. The reaction mixture was dialyzed against DMSO. After evaporation of the solvent, the degree of hydrophobization of all monohydroxyl units (57 mol %; 35 hydrophobic units per macromolecule) based on the ^1^H NMR spectrum recorded in deuterated DMSO-*d*_6_, comparing the integration of methyl protons from acetal groups in terminal units (1.15 and 1.35 ppm) and aromatic protons from phenyl groups (7.21 to 8.02 ppm).

^1^H NMR (400 MHz, DMSO-*d*_6_): δ (ppm) for the BE-HbPGL derivative 7.95-2H, 7.62-1H, 7.50-2H (phenyl), 5.25 (1H-CH-L_1,4-hydrophobic_), 5.01–4.31 (1H-OH-L_1,3_, L_1,4_), 4.24 (1H-CH_2_-L_1,3-hydrophobic_), 4.13 (1H-CH_2_-T_Acetal_), 3.94 (1H-CH_2_-T_Acetal_), 3.82–3.14 (HbPGl-backbone), 1.29 (3H-CH_3_ T_Acetal_), 1.25 (3H-CH_3_ T_Acetal_).

After the determination of the hydrophobization degree, the diol groups were deprotected by adding a 0.1 M HCl aqueous solution to the polymer solution in DMSO and stirred overnight. Finally, the mixture was dialyzed against deionized water and analyzed with ^1^H NMR spectroscopy in deuterated DMSO.

^1^H NMR (400 MHz, DMSO-*d*_6_): δ (ppm) for the BE-HbPGL derivative 7.97-2H, 7.63-1H, 7.52-2H (phenyl), 5.26 (1H-CH-L_1,4-hydrophobic_), 4.93–4.33 (1H-OH-L_1,3_, L_1,4_, T), 4.25 (1H-CH_2_-L_1,3-hydrophobic_), 4.01–2.98 (HbPGL-backbone).

### 2.5 Preparation of Viscous Liquid Drug Formulation

A series of HbPGL solutions with a core enriched in 4-biphenylurethane groups (0.065 g) were prepared and dissolved in 1 mL of methanol. CLT in the amounts of 12.2, 23.0, 32.5, or 41.5 mg, respectively, was dissolved in 5 mL of methanol to obtain a series of drug solutions. In the next step, the polymer and drug solutions were mixed and stirred for 30 min. Methanol was then evaporated completely at 40 °C. Upon confirmation of complete removal of methanol, a series of transparent formulations were obtained, *i.e.*, mixtures of polymer and drug, to which 131 or 262 μL of deionized water was added and mechanically mixed. The chemical composition of the aqueous drug formulations of internally hydrophobized HbPGL with the phenyl and 4-biphenyl groups is presented in Table 1.

**Table 1:** Structural Characteristics of the Aryl-Enriched HbPGLs

hydrophobized HbPGL	molar fraction of hydrophobized constitutional units bearing monohydroxyl groups (L_13_ + L_14_)	number of hydrophobized units per one macromolecule
BE57	57	35
PC66	66	41
BPh40	40	24

### 2.6 Rheology

Flow curves of prepared formulations were investigated at 25 and 37 °C on a MARS 40 rheometer (Thermo Scientific HAAKE) in the range of shear rate from 0.1 to 20.0 s^–1^.

Temperature sweep tests of formulations in the range from 4 to 50 °C were performed in the mode of controlled deformation at a strain equal to 0.2% and a frequency of 0.16 Hz using the continuous heating program with a heating rate of 6.5 °C/min.

### 2.7 Differential Scanning Calorimetry, DSC

The thermal properties of the neat BPh-HbPGL copolymer, CLT, and BPh-HbPGL-based CLT formulations were evaluated using a DSC [TA Instruments (2500 Discovery series)]. A specific amount of sample was heated in a sealed aluminum pan from room temperature to 170 °C with a heating rate of 10 °C/min.

### 2.8 Size Exclusion Chromatography

To determine the molecular mass distribution, weight-average molecular mass (*M*_w_) and dispersity (*D̵*) samples were analyzed by using gel permeation chromatography. The SEC system was provided Testa Analytical Solutions (Berlin, Germany) and consisted of an isocratic pump, automatic injector, and set of 2 separation columns (Suprema Lux analytical linear XL column) with Suprema Lux analytical SDV precolumn supplied by PSS Polymer Standards Service GmbH (Mainz, Germany) in a thermostatic column oven, as well as three detectors: multiangle laser light scattering detector (MALLS) (Brookhaven Instruments Corporation, Holtsville, New York, USA), differential refractive index, and differential pressure viscosity detector, the last two combined in a single assembly with common sample path (Testa Analytical Solutions, Berlin, Germany). As an eluent, 0.1 M NaNO_3_ was used, the flow rate and temperature were maintained at 1 mL min^–1^ and 30 °C, respectively, and the injection loop volume was 100 μL. Data were collected and processed with ParSEC SEC software (Brookhaven Instrument Corporation, Holtsville, New York, USA). All measurements were conducted in triplicate.

### 2.9 FT-IR Measurements

The FTIR spectra were measured on a Thermo Scientific Nicolet iS50 spectrometer using an ATR accessory equipped with a diamond crystal. The spectra were measured with a resolution of 2 cm^–1^, and 64 scans were averaged to achieve a high signal-to-noise ratio, allowing for further mathematical treatment of the results. The polynomial baseline was extracted from each spectrum, and then the spectra were normalized. The normalization procedures were selected depending on the problem to be solved and are described in detail with the presentation of the results. Difference spectra were calculated in relation to spectra of the dry polymer, dry polymer with an encapsulated drug, polymer solution, or deionized water.

### 2.10 Skin Permeability Investigations

A formulation constructed of drug-loaded hydrophobized HbPGL containing equivalent 0.9 mg of CLT was placed on the Strat-M membrane into a donor compartment of the Franz cell. For comparison, Clotidal Max, CLT 500 mg commercially available vaginal tablet, USP Health, and the suspension of pure CLT in the deionized water were investigated at the same drug concentration in the analyzed samples.

The receptor compartment was filled with 12.5 mL of simulated vaginal fluid at 37 ± 1 °C. Aliquots of 0.5 mL of the receptor solution were taken at different time intervals for around 145 h. Each withdrawn aliquot was immediately replaced with the same volume of the corresponding fresh portion of the simulated vaginal fluid.

CLT concentrations were determined by an ACQUITY UPLC I-Class chromatography system coupled with a SYNAPT G2-Si mass spectrometer equipped with an electrospray source and quadrupole time-of-flight mass analyzer (Waters Corp., Milford, MA, USA). An Acquity BEH C18 column (100 × 2.1 mm, 1.7 μm) maintained at 45 °C was used for the chromatographic separation of an analyte. The mobile phase was prepared by mixing 0.1% formic acid (A) and 0.1% formic acid in acetonitrile (B). The elution gradient was: 32% B (0–1.0 min), 32–95% B (1.0–3.0 min), 95–95% B (3.0–3.5 min), 95–32% B (3.5–3.52 min), and 32–32% B (3.52–7.0 min). The flow rate was 0.45 mL/min, and the injection volume was 0.5 μL for CLT.

For mass spectrometric detection, the electrospray source was operated in a positive resolution mode. The optimized source parameters were: capillary voltage of 3.0 kV, cone voltage of 20 V, desolvation gas flow of 400 L/h at a temperature 350 °C, nebulizer gas pressure of 6.5 bar, and source temperature of 100 °C. Mass spectra were recorded over an *m*/*z* range of 100 to 1200. Mass spectrometer conditions were optimized by direct infusion of a standard solution. The system was controlled using MassLynx software (Version 4.1), and data processing (peak area integration and construction of the calibration curve) was performed by the TargetLynx software.

The initial stock calibration solution of CLT was created in simulated vaginal fluid. The stock solutions were serially diluted with 0.5 mL of simulated vaginal fluid and 0.5 mL of methanol to obtain working solutions at several concentration levels. The calibration curves were prepared at ten different concentrations of drug solutions and were linear in a concentration range from 0.1 to 20.0 μg/mL for CLT with a correlation coefficient of >0.995.

### 2.11 Antifungal Activity

The antifungal activity was investigated against *C. albicans* ATCC 90028 and *C. glabrata* ATCC 2001. Fungal cultures were stored at 4 °C and subcultured once a month. Prior to the inoculation of the strains with analyzed compounds containing CLT, the fungal strains were grown at 35 °C on Sabouraud agar.

Antifungal activity was evaluated by the disk diffusion assay according to the European Committee on Antimicrobial Susceptibility Testing (EUCAST). The fungal reference strains, such as *C. albicans* ATCC 90028 and *C. glabrata* ATCC 2001, were cultured on Sabouraud dextrose agar and incubated at 35 °C for 24 h. A single colony from an overnight fungal culture plate was seeded into 5 mL of an appropriate prewarmed growth medium broth (Sabouraud). Next, the cell density of the inocula was adjusted using a validated autocalibrated turbidimeter, assuring a 0.5 McFarland standard. Then the suspension was diluted 1:10 in sterile distilled water to yield (1–5) × 10^5^ CFU/mL. Using a sterile swab, cultures were spread evenly onto prewarmed 37 °C Sabouraud agar plates. Finally, the different disks (diameter 5 mm), previously impregnated with formulations E, M containing 1400 μg of CLT, and J containing 480 μg of CLT were placed in triplicate in the inoculated agar, and the dishes were left in an incubator at 37 °C. Standard disks of 480 and 1400 μg of tablet (Clotidal Max, CLT 500 mg vaginal tablet, USP Health) were used as a positive control. After 16, 24, and 42 h of incubation, in the case of J gel, additionally, after 7 days, the diameters of the inhibition zone presented by the tested substances were measured in millimeters, which are reported as inhibition halos.

## 3 Results and Discussion

### 3.1 Preparation of Aqueous Formulations of CLT with Aryl-Enriched HbPGLs

For our investigations, we applied HbPGL (*M*_w_ = 12,000) of significantly hydrophobized monohydroxylated units present in the linear constitutional units, *i.e.*, L_13_ and L_14_ (Scheme 1), with benzoyl chloride, phenyl isocyanate, and biphenyl isocyanate at the degree equal to 57, 66, and 40 mol %, respectively, obtaining the following BE57, PC66, and BPh40 HbPGL derivatives (Table 1).

Our previous studies have shown that for the formation of hydrogels based on CLT-loaded HbPGL in monohydroxylated units that were hydrophobized at lower degrees, and suitable as drug carriers for intravaginal therapy, the usage of the second cross-linkable component was needed.^22^ The encapsulation of CLT in highly hydrophobized HbPGLs leads to an evident increase of the viscosity. Therefore, here, we focus on the explanation of cross-linking mechanisms observed for highly aryl-enriched HbPGLs *via* CLT encapsulation and acquaintance with the knowledge of the characteristics of the obtained drug formulations, such as the rheological properties, and antifungal activity, along with the determination of the CLT state. Understanding the nature of these constructs is extremely important because of their high potential for biomedical applications due to their simplicity of composition.

The hydrophobization of the HbPGL core required the protection of 1,2-diol moieties of terminal units (35 mol % of all repeating units) to avoid the modification of the HbPGL shell. It was achieved by the protection of diol groups in the form of acetals in the reaction with solketal catalyzed with PTSA. The protection of terminal units was verified using ^13^C INVGATE NMR spectroscopy to ensure that the hydrophobization of HbPGL was performed in the core of macromolecules. The detailed spectroscopic analysis of each hydrophobized HbPGL at each reaction step is given in the Supporting Information (Figures S1–S12).

### 3.2 Preparation of Aqueous Drug–Polymer Formulations

**Figure 1:**
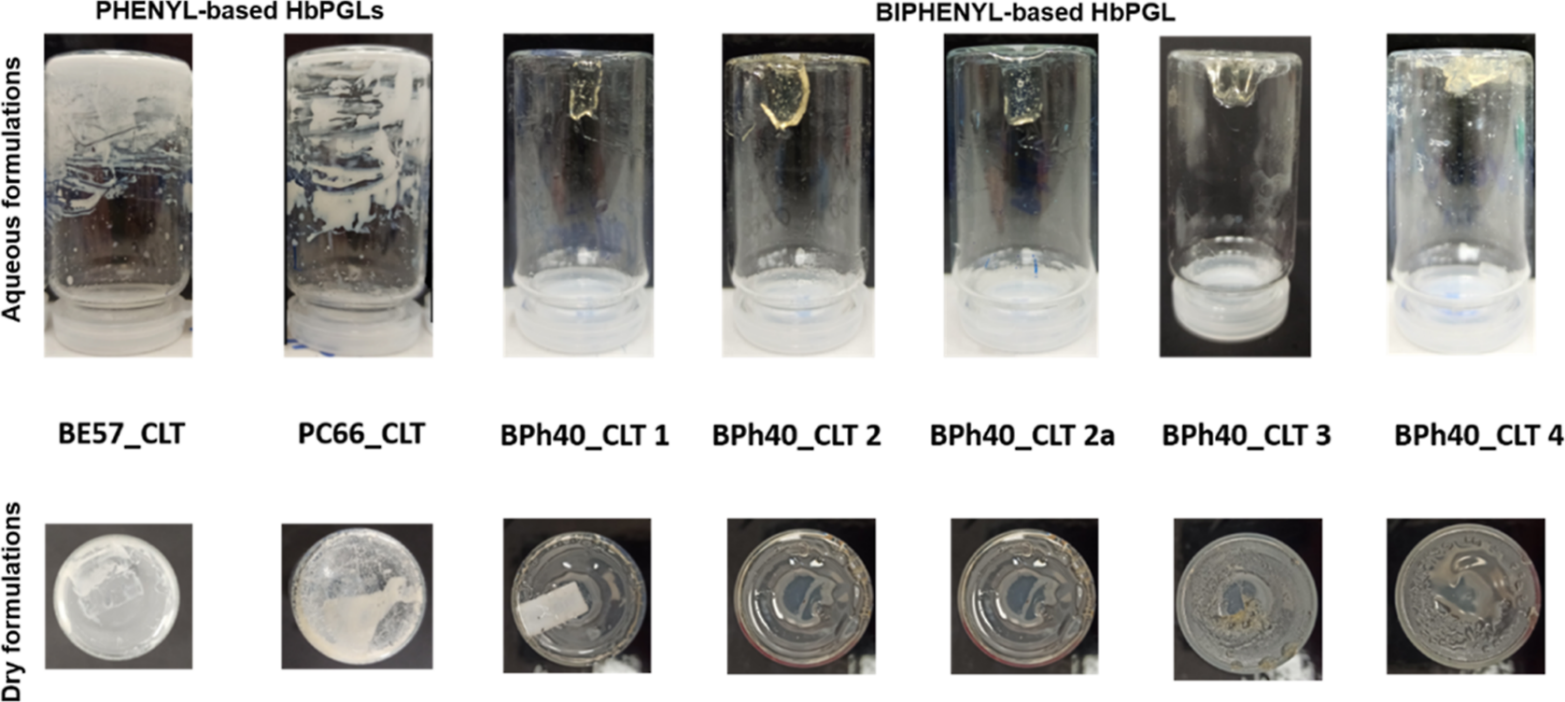
Pictures of the aqueous formulations of clotrimazole with internally aryl-enriched hyperbranched polyglycidols.

CLT was encapsulated within aryl-enriched HbPGL using a solvent evaporation method^23^ using methanol as a good solvent for both drug and polymer. Among all investigated formulations, a molar ratio of drug/macromolecule equal to 32 was optimal to obtain uniform gel-like structured fluids for all HbPGL derivatives of significantly reduced flow properties. The phenyl-HbPGL-based formulations were opaque (Figure 1). Surprisingly, the BPh40 derivative formed a homogeneous and transparent formulation (Figure 1) at the abovementioned drug–polymer molar ratio. The transparency of BPh40-based formulation inputs that the applied polymer matrix is compatible with CLT, improving its solubility in the aqueous medium. In addition, we prepared the BPh40-based formulations by applying a lower molar fraction of drug molecules to a macromolecule (Table 2). Despite the significant reduction of the molar fraction of the drug in the formulations, the obtained systems also displayed a gel-like character. Even the significant dilution of both the polymer and drug (Table 2) resulted in the formation of a stable gel-like formulation.

**Table 2:** Chemical Composition of the Aqueous Drug Formulations of Internally Hydrophobized Hyperbranched Polyglycidol with Phenyl and 4-Biphenyl Groups

formulation	molar ratio of CLT to *h*HbPGL	, g/mL	, g/mL
BE57_CLT	32	0.496	0.317
PC66_CLT	32	0.496	0.317
BPh40_CLT_1	8	0.496	0.093
BPh40_CLT_2	16	0.496	0.176
BPh40_CLT_2a	16	0.248	0.088
BPh40_CLT_3	24	0.496	0.248
BPh40_CLT_4	32	0.496	0.317

### 3.3 Rheological Characterization of Aqueous Drug–Polymer Formulations

**Figure 2:**
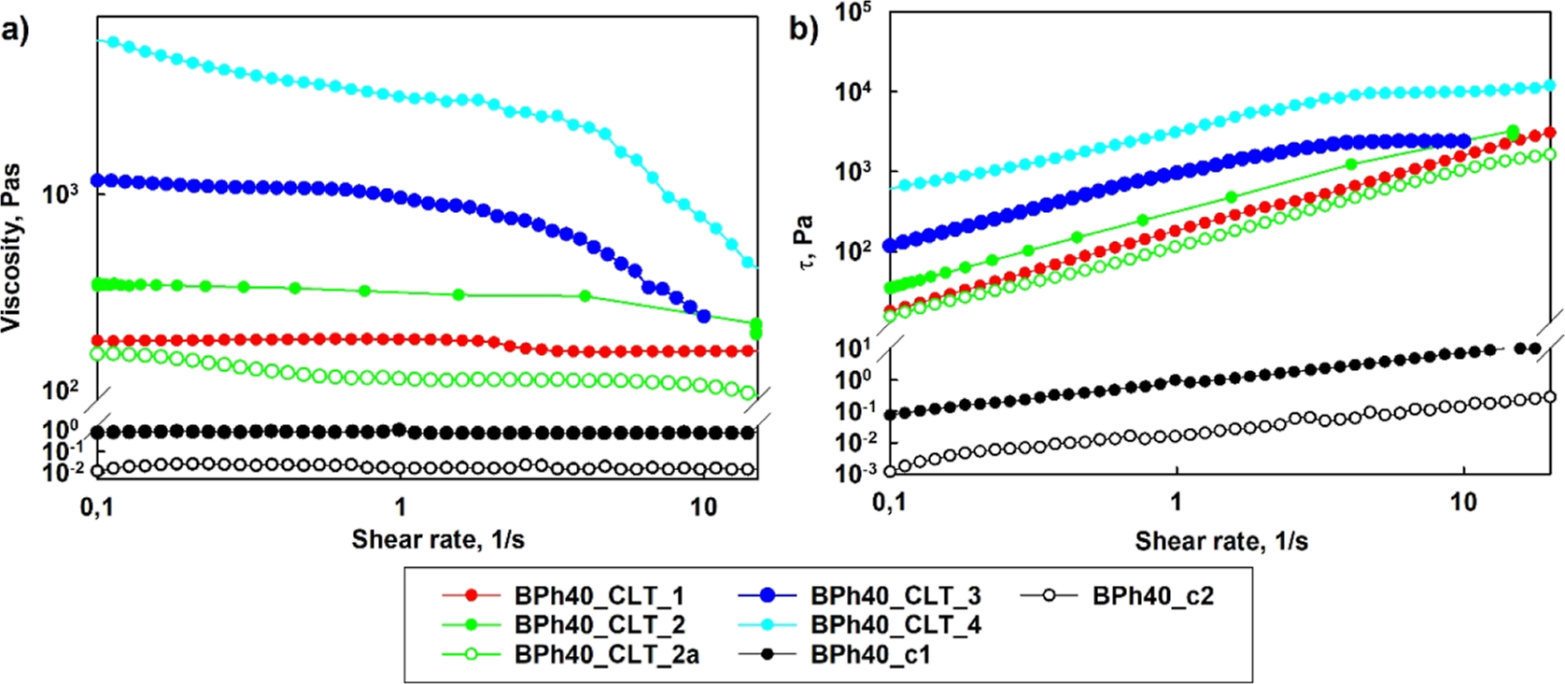
Dependence of viscosity (a) and stress (b) on the shear rate recorded at 37 °C for the aqueous solutions of BPh40 and aqueous formulations prepared of clotrimazole-loaded BPh40.

Aqueous solutions of the neat copolymers BE57, PC66, and BPh40 at a concentration of 500 mg/mL are low-viscosity Newtonian liquids (Figures 2 and S13). The incorporation of CLT within hydrophobized HbPGL derivatives (drug–polymer molar ratio = 32) *via* a solvent evaporation method resulted in an evident reduction of the flow properties of all hydrated drug–polymer formulations as an effect of the increased viscosity.

Rheological investigations of aqueous formulations of CLT with HbPGL internally hydrophobized with aryl moieties, *i.e.*, the benzoyl (BE57), phenylurethane (PC66), and 4-biphenylurethane (BPh40) groups, respectively, revealed the non-Newtonian behavior (Figures 2 and S13). Obtained aqueous clotrimazole–*h*HbPGL formulations are structural fluids, *i.e.*, pseudoplastic or Bingham fluids.^24,25^

It is noteworthy that all aqueous CLT formulations with aryl-based HbPGLs are shear-thinning (Figures S13, 2, and 3), which means that the viscosity of each fluid decreases with the increasing shear rate. The rheological properties of the liquid formulations indicated that they can be injected into the target site, and therefore these systems seem to be suitable for vaginal use, ensuring easy distribution of the preparation on the surface of the affected tissue.^26^

**Figure 3:**
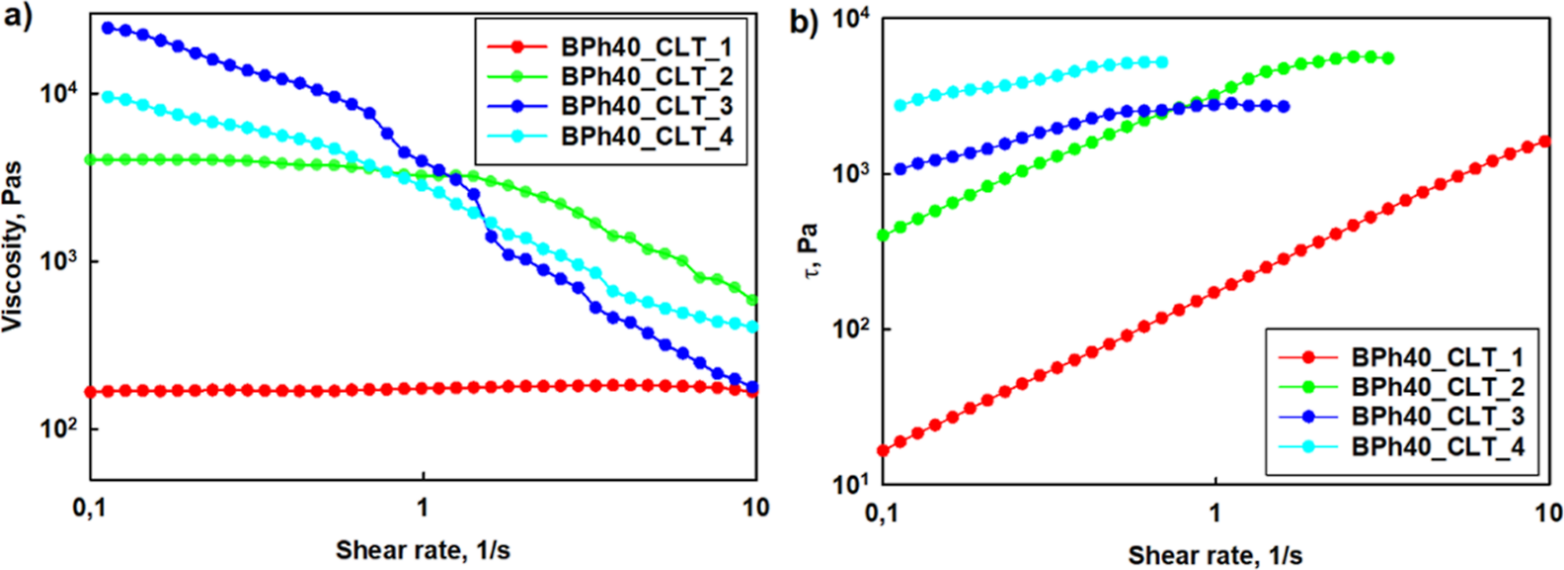
Dependence of viscosity (a) and stress (b) on the shear rate recorded at 25 °C for the aqueous solutions of BPh40 and aqueous formulations prepared with clotrimazole-loaded BPh40.

In the case of phenyl-enriched HbPGL derivatives, a higher increase in the viscosity of the aqueous solution was observed for PC66 in comparison to that for BE57 (Figure S13). Zero shear viscosity (η_0_), determined by the extrapolation of the viscosity *vs* shear rate dependence to the shear rate equal 0, was equal to 0.08 and 0.33 Pa s for the aqueous formulations based on BE57 and PC66 derivatives, respectively (Figure S13). Encapsulation of CLT led to an increase of viscosity to 0.77 and 49.50 Pa s for BE57_CLT and PC66_CLT, respectively. Compared to phenyl-enriched HbPGL derivatives, the increased viscosity effect of the aqueous solution of biphenyl-modified HbPGL upon CLT loading (at the same drug–polymer molar ratio) was even stronger, despite the lower degree of hydrophobization of monohydroxylated HbPGL units. These data indicate that for biphenyl-modified HbPGL, the intermolecular cross-linking ability of internally hydrophobized HbPGL macromolecules with CLT was enhanced.

It could be concluded that, in addition to the effect of CLT on the structural properties of the fluids obtained, the size of the aryl group incorporated into the core plays a key role in the formation of structured fluids. In addition, it seems that the higher the molar fraction of monohydroxylated units remaining intact, the higher the fraction of absorbed water. We demonstrated that the usage of a lower amount of drug for the preparation of the BPh40 formulation, *i.e.*, BPh40_CLT_2, resulted in the formation of a stable, transparent drug carrier. In contrast to phenyl-based HbPGL derivatives, HbPGL hydrophobized with biphenyl moieties was able to maintain water in the formulation despite the decrease of the drug weight to water volume ratio, *i.e.*, from 0.317 g/mL (BPh40_CLT_4) to 0.093 g/mL (BPh40_CLT_1), respectively. The decrease in the concentrations of fluid components, *i.e.*, both polymer and drug, at the same drug–polymer ratio (BPh40_CLT_2a), resulted in the formation of a stable homogeneous formulation but of a lower viscosity. It has been shown that it is easier to obtain an optimal composition of the drug formulation. In addition, in contrast to the BPh40-CLT formulation, phase separation was observed for phenyl-HbPGL derivative-based drug formulations over long-time storage.

This extended stability makes biphenyl-HbPGL a very promising polymer for the construction of two-component CLT formulations. To demonstrate the effect of CLT on the behavior of aryl-modified HbPGL macromolecules in aqueous solutions, we prepared a series of formulations based on BPh40 in a constant polymer fraction, however, differing in the molar ratio of drug to polymer from 8 (BPh40_CLT_1) to 32 (BPh40_CLT_4). Zero shear viscosity of drug formulations based on BPh40 gradually increased from 183, 353, to 1080 Pa s at body temperature for formulations with the drug at a molar fraction as follows: 8, 16, and 24, respectively (Table 3).

**Table 3:** Zero Viscosity Values (η_0_) of Formulations Based on Clotrimazole-Loaded Aryl-Modified Hyperbranched Polyglycidols with the Indication of a Presence of the Yield Point (τ_c_) Determined at 37 °C^a^

aqueous formulation	molar ratio of CLT to copolymer	η_0_, Pa s (37 °C)	τ_c_, Pa (37 °C)
BE57_c1	0	0.08	
PC66_c1	0	0.33	
BPh40_c1	0	0.79	
BPh40_c2	0	0.03	
BE57_CLT	32	0.77	
PC66_CLT	32	49.50	
BPh40_CLT 1	8	183	
BPh40_CLT 2	16	353	2.72 ± 0.22
BPh40_CLT 2a	16	156	2.16 ± 0.45
BPh40_CLT 3	24	1080	13.62 ± 0.49
BPh40_CLT 4	32		329 ± 5.0

a BE57_c1 = PC66_c1 = BPh40_c1 = 500 mg/mL; BPh40_c2 = 250 mg/mL.

The dependence of stress (τ) on the shear rate revealed that the properties of the drug formulation can be modulated not only with the amount of drug used in the formulation but also on thermal conditions (Figures 2b, 3b, and S13 and Tables 3 and S1). Such CLT formulations as BPh40_CLT 2, BPh40_CLT 3, and BPh40_CLT 4 exhibit the yield point at room temperature, which was equal to as follows: 76, 653, and 2363 Pa. At 37 °C, the yield point decreases to 2.7, 13.6, and 329 Pa, respectively. The CLT fluid formulations showing the yield point are Bingham fluids. The other formulations based on aryl-hydrophobized HbPGLs behave as pseudoplastic fluids.

It is noteworthy that in the case of formulations being Bingham fluids at 37 °C, their flow can be merely triggered by exceeding the stress of the yield point, *i.e.*, a critical stress value that is needed to break the structure. It is assumed that uncontrolled release of the drug formulation upon administration in the vagina is impossible, as is typical for used gynecological suppositories. The viscosity of other formulations, *i.e.*, fluids that did not exhibit the yield point, is high enough to maintain the drug carrier in the vagina. For example, the flow rate of the BPh40_CLT 2a formulation (zero shear viscosity ≈ 156 Pa s) of 2 mm thickness and 5 cm wide flowing on the surface inclined at θ = 90° to the horizontal is approximately 1.2 μg_fluid_/h.

The rheological properties of drug carriers based on the structured fluids point out that they can be maintained longer in the afflicted area of the vagina compared to traditional suppositories, which are required to be administered very often, as their content is very often removed in an uncontrolled manner. The prolonged residence time of an active substance with a diseased site is of great importance in ensuring the transport of drug molecules and thus increasing the efficiency of therapy.

**Figure 4:**
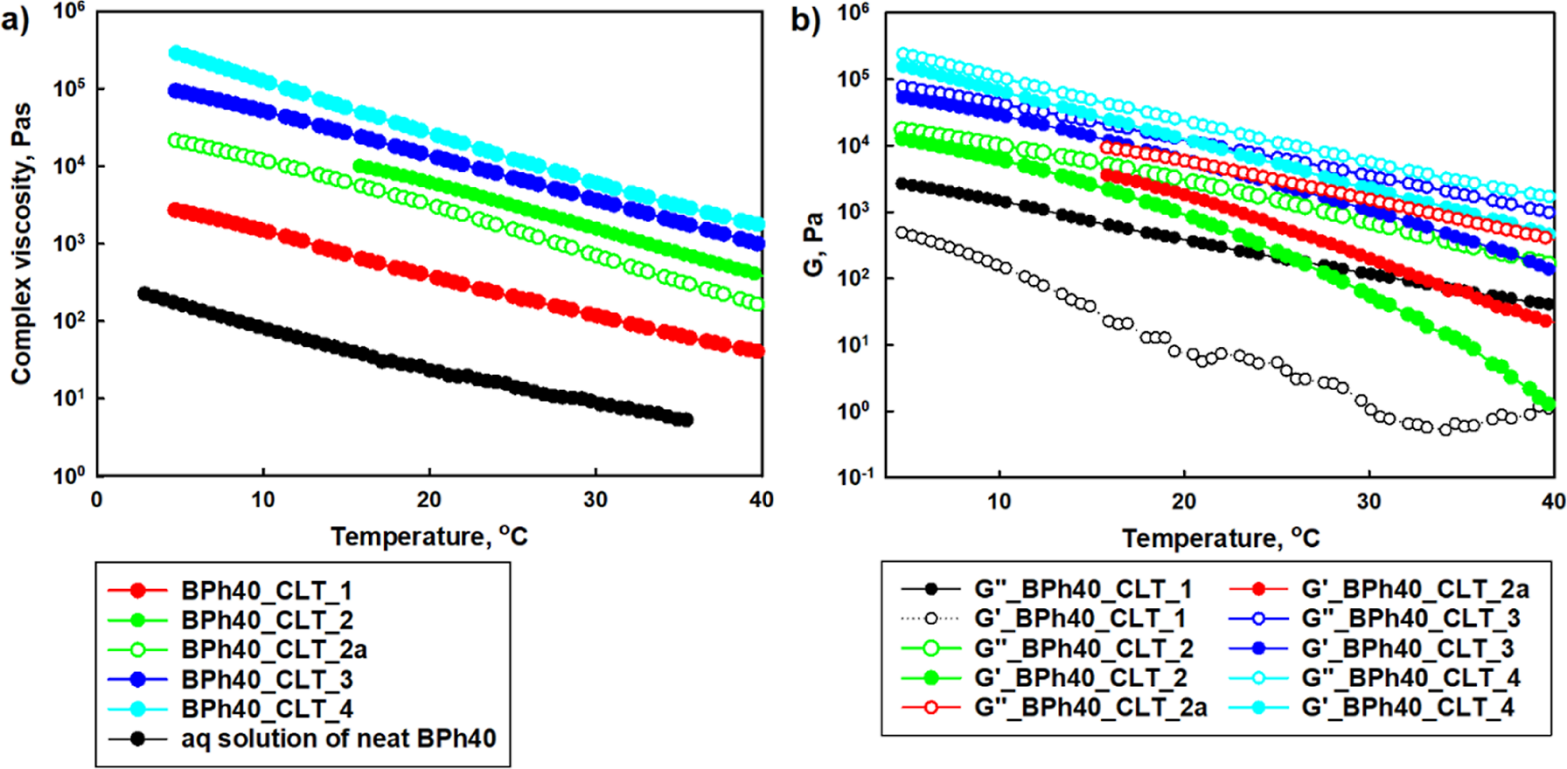
Dependence of storage (*G*′) and loss modulus (*G*″) (a) and complex viscosity (b) on the temperature recorded for the aqueous formulations prepared of clotrimazole-loaded BPh40.

A temperature sweep study performed for biphenyl-based formulations revealed the dominant nature of viscous properties in a wide range of temperatures (*G*″ > *G*′), Figure 4. Based on the fact that the viscosity of each formulation decreased with the increasing temperature, we stated that the CLT formulation with biphenyl-modified HbPGL displayed thermosensitive behavior. However, due to the fact that the slope of the dependence of the complex viscosity *versus* temperature determined for the neat BPh40 is similar compared to its formulation with the drug, it can be concluded that the thermosensitive character is governed by intra- and intermolecular hydrogen bonds in which the polymer itself is involved.

### 3.4 FT-IR Study of Structured Fluids Based on CLT-Loaded Hydrophobized HbPGLs

To evaluate the distribution of CLT in internally aryl-enriched HbPGLs, we performed a detailed FTIR analysis, recording spectra of dry copolymers, dry CLT–copolymer mixtures, and their aqueous formulations, along with spectra of CLT in the crystalline form and dissolved in methanol. The FTIR spectrum of CLT in crystalline form differs significantly from that of CLT dissolved in methanol, *e.g.*, molecularly dispersed (Figure S14). We distinguished the difference in the spectra of CLT in the crystalline and in the dissolved state in the range of the wavenumbers from 720 to 780 cm^–1^, *i.e.*, for crystalline CLT, a triple-band was observed in this range, whereas for dissolved CLT, a single broad band was typical. We established that subtracting the FTIR spectrum of the hydrophobized polymer from that of its mixture with the drug yields the extracted CLT spectrum, which can provide information about the state of the drug molecules in the investigated formulations, *i.e.*, whether drug molecules inside the polymer carrier exist in crystalline form or are molecularly dispersed like in the solution. This approach can provide knowledge about the compatibility of the polymer with CLT. The extraction of the spectrum obtained for the aqueous solution of copolymer from the spectrum of the aqueous drug–copolymer formulation allows evaluation of the CLT state in the aqueous polymer-based formulations.

**Figure 5:**
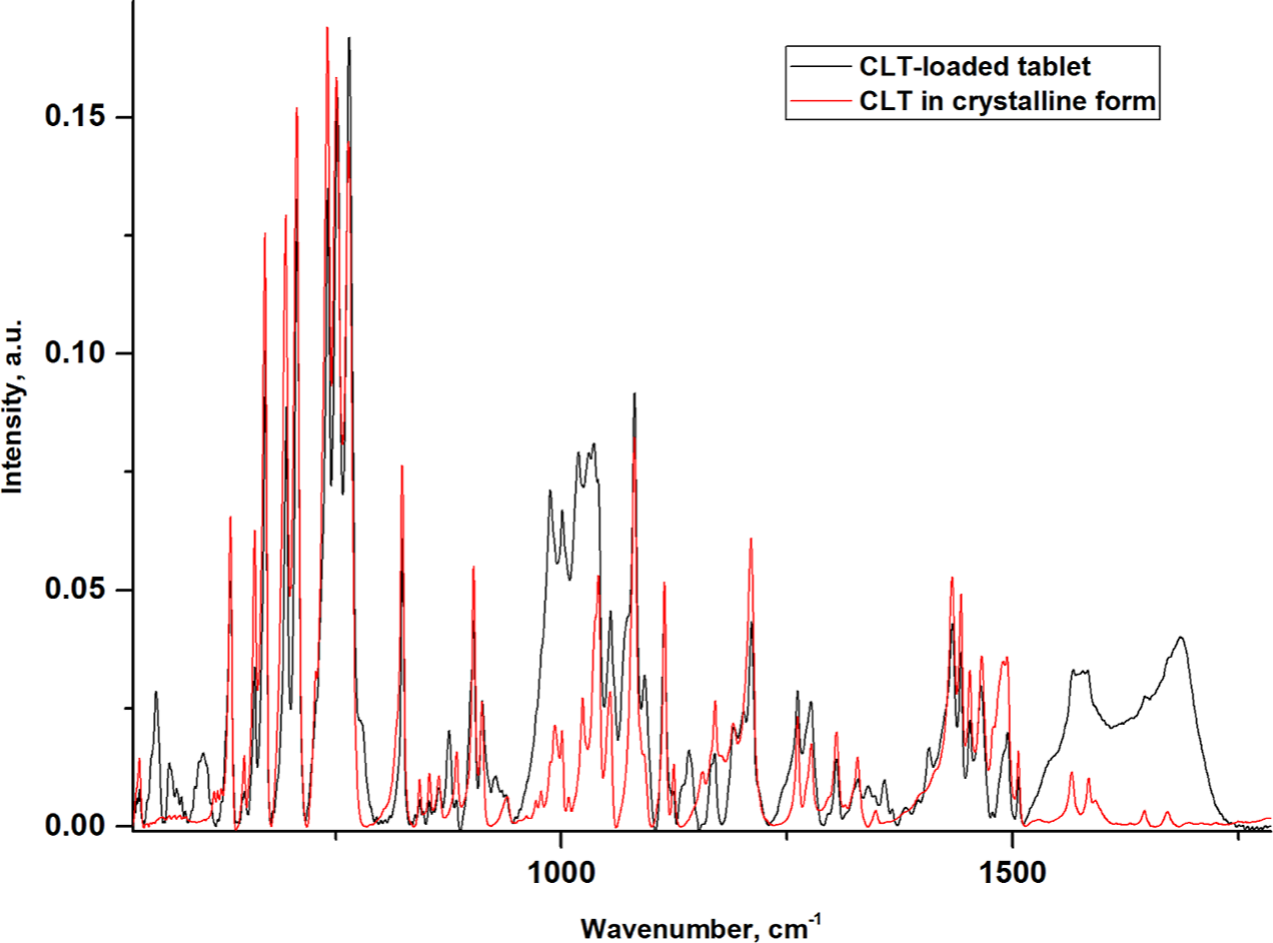
Comparison of the FTIR spectrum of a commercially available clotrimazole-loaded tablet (Clotidal MAX) and the FTIR spectrum collected for clotrimazole in the crystalline state.

**Figure 6:**
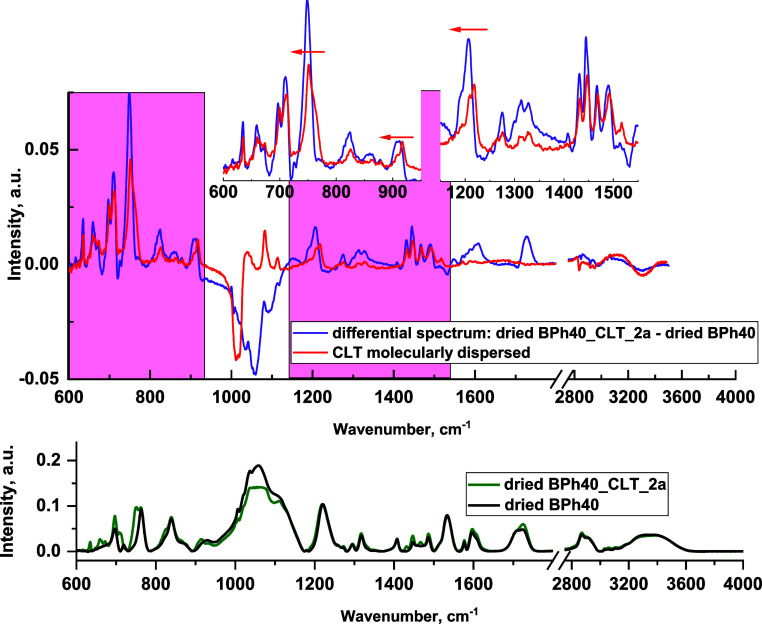
Comparison of FTIR spectra of dried, neat BPh40, and BPh40 loaded with clotrimazole (bottom). Differential spectrum of the FTIR spectra is shown in the bottom chart compared with the FTIR spectrum of clotrimazole in a molecularly dispersed state. Spectral regions where the clotrimazole lines sensitive to intermolecular interactions occur are zoomed in insets.

A comparison of the FTIR spectrum in the range of wavenumbers from 720 to 780 cm^–1^ of commercially available formulations containing CLT (Clotidal MAX) and the spectrum collected for CLT in powder (Figure 5) confirmed that the drug formed crystals in both. The FTIR differential spectra obtained for CLT formulations based on phenyl-HbPGLs, *i.e.*, BE57 (Figure S15) and PC66 (Figure S16) copolymers, also revealed that CLT occurs in the polycrystalline state in the matrix of both in dry and aqueous states. Despite the significant hydrophobization degree of internal monohydroxyl groups of HbPGL with the phenyl groups incorporated *via* ester (57 mol %, 35 of hydrophobized units per macromolecule) or urethane bonds (66 mol %, 41 of hydrophobized units per macromolecule), these amphiphilic constructs are not promising as solubilizing matrices for poorly water-soluble CLT. CLT formulations based on phenyl-enriched HbPGLs were opaque in both dry and aqueous conditions, which confirmed the lack of phenyl-enriched HbPGL compatibility with the drug in the investigated molar ratio of the drug to polymer, *i.e.*, 32:1. CLT formulations based on biphenyl-enriched HbPGL derivatives, that is, HbPGL whose monohydroxylated units in the core were hydrophobized to a lower degree, were transparent at room temperature regardless of the molar ratio of drug molecules per one macromolecule, *i.e.*, 8:1, 16:1, 24:1, and 32:1. Subtraction of the spectrum of biphenyl-enriched HbPGL from that of its mixture with CLT performed for dried BPh40_CLT_2a and BPh40_CLT_4 mixtures provided a spectrum of CLT characteristic for its well-solubilized form, *i.e.*, such as that obtained in the methanolic solution (Figures 6 and S17). Undoubtedly, it indicates that CLT was molecularly distributed in the dry BPh40 matrix despite the amount of encapsulated drug in the broad range of concentrations.

The comparison of the FTIR spectrum of CLT obtained after subtraction of the spectrum of methanol from the spectrum of CLT loaded in the dry BPh40 carrier shows very good agreement, and only the shifts are observed within the bands located around 750, 920, and 1200 cm^–1^. These lines may be assigned to the stretching of C–Cl groups (750 cm^–1^)^7^ and C–H and C–N groups in the imidazole ring (917 and 1206 cm^–1^, respectively).^27^ These lines are sensitive to interactions with the environment. Their shift is indirect evidence of the interaction of the polymer carrier with the drug through halogen bonds and probably through the imidazole ring. The shift toward lower wavenumbers of the bands originating from the C–Cl bond (750 cm^–1^) is typical for the formation of halogen bonds between Cl and a nucleophile in the system (O, N, or Ph).^28,29^ The other CLT bands are located at the same wavenumbers independently of the environment, which confirms that the signal processing used is correct.

To study the behavior of BPh40 in the formulation with the drug, the vibrational spectra of dry BPh40 and dry BPh40 loaded with CLT were compared (Figure 6). The observed changes, *i.e.*, a shift of the bands 1540 and 1750 cm^–1^ (combination of N–H bending with C–N stretching and C=O stretching, respectively^30,31^) toward higher wavenumbers indicate the breaking of intramolecular hydrogen bonds. Moreover, a shift of the band around 1220 cm^–1^ (C–O–C stretching^30,31^) toward lower wavenumbers confirms the breaking of hydrogen bonds and the transfer of charge toward the chain, thus shortening the C=O bonds and lengthening the C–O–C located next to it.^32,33^

In the next step, we focused on the behavior of both CLT and BPh40 in their aqueous formulations. The position of the bands assigned to CLT in the polymer formulation is independent of the presence of water in the environment. This proves that CLT molecules remain bound to the polymer carrier after the addition of water. Moreover, these aqueous drug formulations were stable without precipitated CLT being detected with the naked eye.

**Figure 7:**
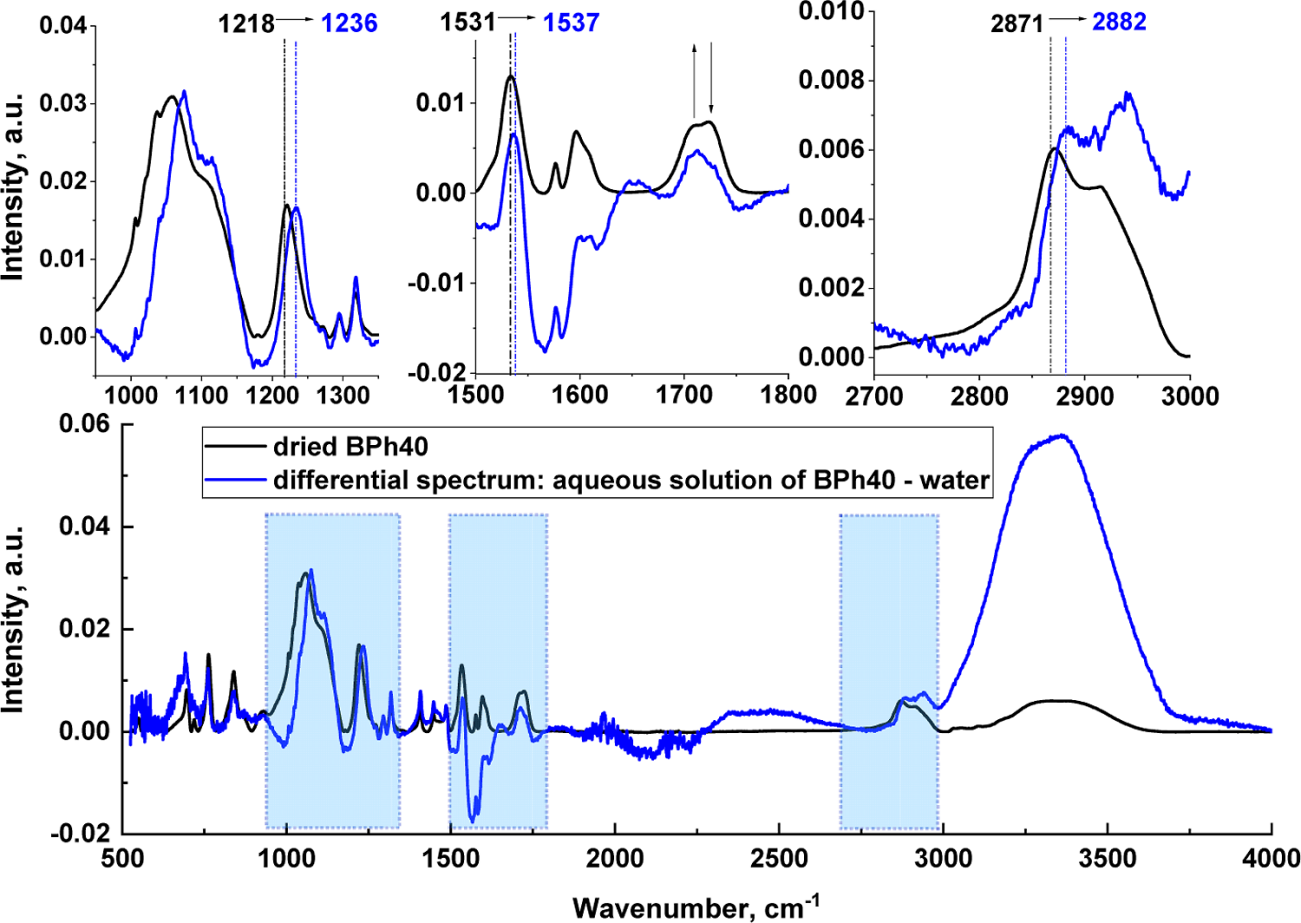
FTIR spectrum of BPh40 in dry and hydrated states. Insets show the spectral regions where the bands of the BPh40 polymer are sensitive to hydration.

To monitor the polymer hydration, a comparison of the normalized FTIR absorption spectrum of neat BPh40 and the differential spectrum of its aqueous solution (the spectrum obtained by the extraction of the water spectrum from the spectrum related to the BPh40 aqueous solution), as shown in Figure 7 was needed. Vibrations of highly polar groups, such as ether or carbonyl groups, should be the most sensitive to water. Their characteristic bands in the FTIR spectrum of neat BPh40 occur at 1218 (stretching of –C–O–C– groups), 1531 (combination of bending of N–H and C–N stretching vibrations), and 1750 cm^–1^ (stretching of C=O group).^30,31^ The first two signals shift toward higher wavenumbers, *i.e.*, to 1236 and 1537 cm^–1^, respectively, which can be explained by the interaction of the ether and amide groups with water *via* hydrogen bonds.^30,31^ These observations are confirmed by the changes observed in the spectral regions related to the stretching of the C=O groups and the stretching of the CH_*x*_ groups (spectral region 2800–3000 cm^–1^). In the first mentioned spectral region, changes in the relative intensities of bands located at 1710 cm^–1^ related to C=O groups involved in the H-bond formation and 1725 cm^–1^ assigned to free C=O groups, *i.e.*, which are not involved in the H-bonding, occur. In the high-wavenumber region, the so-called improper blue shift of line 2871 cm^–1^ (to 2882 cm^–1^) is observed. This effect has been discussed previously.^33−35^

For comparison, the FTIR spectra of BE57 and PC66 in both dry and hydrated states are given in Figures S18 and S19, respectively. In the case of BE57, its characteristic bands in the FTIR spectrum occur at 1272 (stretching of C–O–C) and 1713 cm^–1^ (stretching of C=O group).^36^ The first signal shifts toward higher wavenumbers (1277 cm^–1^), which can be explained by the interaction of the ether groups with water *via* hydrogen bonds. This observation is confirmed by the changes observed in the spectral regions related to the stretching of the C=O groups and the stretching of CH_*x*_ groups (spectral region 2800–3000 cm^–1^). Analysis of the second characteristic band shows changes in the relative intensity of bands located at 1667 cm^–1^ assigned to C=O groups involved in the H-bond formation and at 1713 cm^–1^ characteristic for free C=O groups. The intensity of the first line increases, while the intensity of the second line decreases slightly. The FTIR spectrum of PC66 does not differ significantly from the FTIR spectrum of BPh40. The characteristic bands for phenylurethane derivative occur at 1220 (stretching C–O–C groups), 1544 (combination of bending of N–H and C–N stretching vibrations), and 1720 cm^–1^ (stretching of C=O group). In the case of PC66, the hydrogen bond formation between ether and amide groups with water is also, as mentioned above, proved by signals shift toward higher wavenumbers (1230 and 1548 cm^–1^). Additional confirmation of this interaction is shown by the changes observed in the spectral region related to the stretching of the C=O groups and the stretching of CH_*x*_ groups (spectral region 2800–3000 cm^–1^). Also, changes in relative intensities of bands located at 1708 cm^–1^ assigned to C=O groups involved in hydrogen bond formation and 1723 cm^–1^ ascribed to free C=O groups, which are not involved in H-bonding, confirm the presence of interaction between water and polymer.

In the final step, the spectrum of the aqueous formulation BPh40_CLT_2a with the spectra of the hydrated polymer, water, and dry BPh40 was compared (Figure S20). Such an approach allowed us to evaluate how the presence of the hydrophobic drug in the polymer structure influenced polymer hydration. The bands visible in the spectrum of the aqueous drug–polymer formulation at 1540 cm^–1^ (assigned to N–H and C–N groups) and 1240 cm^–1^ (C–O–C groups) are significantly shifted toward higher wavenumbers in relation to neat BPh40, which evidenced their good hydration.^32,33^ However, these shifts are smaller than those in the case of the BPh40 aqueous solution. This observation leads to the conclusion that the polymer in the aqueous drug-BPh40 formulation exhibits a more hydrophobic behavior than the polymer itself in water. It means that the CLT interacting with the BPh40 affects the polymer carrier, making it more hydrophobic.

A comparison of the FTIR spectra of the aqueous solution of PC66 with the spectrum of its structured fluid with CLT, water, and dried PC66 is shown in Figure S21. It should be noticed that the bands sensitive to polymer hydration, namely, lines assigned to C–O–C stretching (at 1220 cm^–1^), combinations of N–H bending with C–N stretching (1540 cm^–1^), and C=O stretching (1720 cm^–1^) occur at the same wavenumbers in the FTIR spectra of both the polymer solution and its structured fluid. It proved that PC66 hydration is not significantly affected by the presence of CLT. It is not surprising, taking into account that the CLT crystallizes inside this carrier. The FTIR spectra of the aqueous solution of BE57, its structured fluid with CLT, water, and dried BE57 were also compared (Figure S22). The structured fluid constructed of BE57 macromolecules exhibited a more hydrophobic behavior in comparison to PC66 and, as a result, contained a smaller water content. Line positions characteristic for C=O and C–O–C stretching (1720 and 1260 cm^–1^, respectively) have maxima at the same wavenumber as for the dry polymer. It proved that BE57 is not well hydrated in the formulation.

The biphenyl-enriched HbPGL, due to the presence of a hydrophobic surface generated by two aryl rings in a single incorporated hydrophobic moiety compared to a single aryl group (in the case of phenyl-modified HbPGLs), was able to interact more effectively with CLT. In addition, a higher molar fraction of intact monohydroxyl groups in the HbPGL core and peripheral diol groups in the macromolecule’s corona made BPh40 able to readily absorb water. From one side, structured fluid formulations based on BPh40 drug mixtures are well-swollen with water, and from another side, the polymer construct ensures effective encapsulation of CLT. It seems that in addition to the urethane group, which is prone to hydrogen bonding, aryl–aryl interactions between aromatic moieties of polymers and drugs can play an additional role in drug–polymer interactions.

Finally, the chemical structure of the polymer carrier significantly affects the ability of the drug to crystallize. Contrary to phenyl-based HbPGL, the biphenyl-based carrier protects CLT against crystallization, which is a strongly needed property, allowing for better drug release control, bioavailability, and, consequently, a better biological response.

This study shows that the balance between the degree of hydrophobization of HbPGL, the hydrophobic nature of the incorporated groups, such as the size of the aromatic surface, and the linkage *via* which a hydrophobic moiety is incorporated is of great importance in obtaining a promising therapeutic formulation.

Given that the solubility of CLT in water is 0.49 μg/mL,^9^ and the concentration of CLT in formulations with BPh40 varied from 0.062 to 0.211 g/mL, assuming that the entire amount of CLT is molecularly dispersed in the formulation, this means that we have increased the solubility of CLT by at least 127,000 times.

### 3.5 DSC Study of Formulations Based on Hydrophobized HbPGL with CLT

The DSC analysis performed for neat CLT showed a sharp endothermic peak at 145.5 °C on first heating, which corresponds to its melting point. Since neat CLT was not able to crystallize at the applied cooling rate, *i.e.*, 10 °C/min, the state of CLT in polymer-based formulations with polymers was investigated in the first heating scan. Thermograms of CLT formulations based on HbPGL hydrophobized with phenyl groups incorporated *via* both ester and urethane bonds revealed the presence of the endothermic peak in the first heating scan starting from room temperature. In comparison to neat CLT, the endothermic peak was broader and shifted to a lower temperature, *i.e.*, 134 °C. DSC thermograms facilitated the determination of the degree of crystallinity of CLT in the formulations based on phenyl-enriched HbPGLs, which is equal to 84 and 93% for benzoyl ester and phenyl urethane HbPGL derivatives, respectively. The lack of an endothermal peak of CLT in DSC thermograms obtained for formulations based on biphenyl-enriched HbPGL confirmed the molecular distribution of encapsulated drug molecules within amphiphilic macromolecules, which is consistent with previously reported results^37^ and the FTIR results shown above. In addition, despite the amount of drug encapsulated within the construct BPh40, *i.e.*, 16 and 32 molecules per macromolecule, the drug was molecularly distributed.

The DSC study confirmed the enhanced solubilization of CLT in the hydrophobized HbPGLs enriched with aryl groups; however, the size of the biphenyl moiety was crucial in the proper distribution of drug molecules within the amphiphilic HbPGL’s construct.

**Figure 8:**
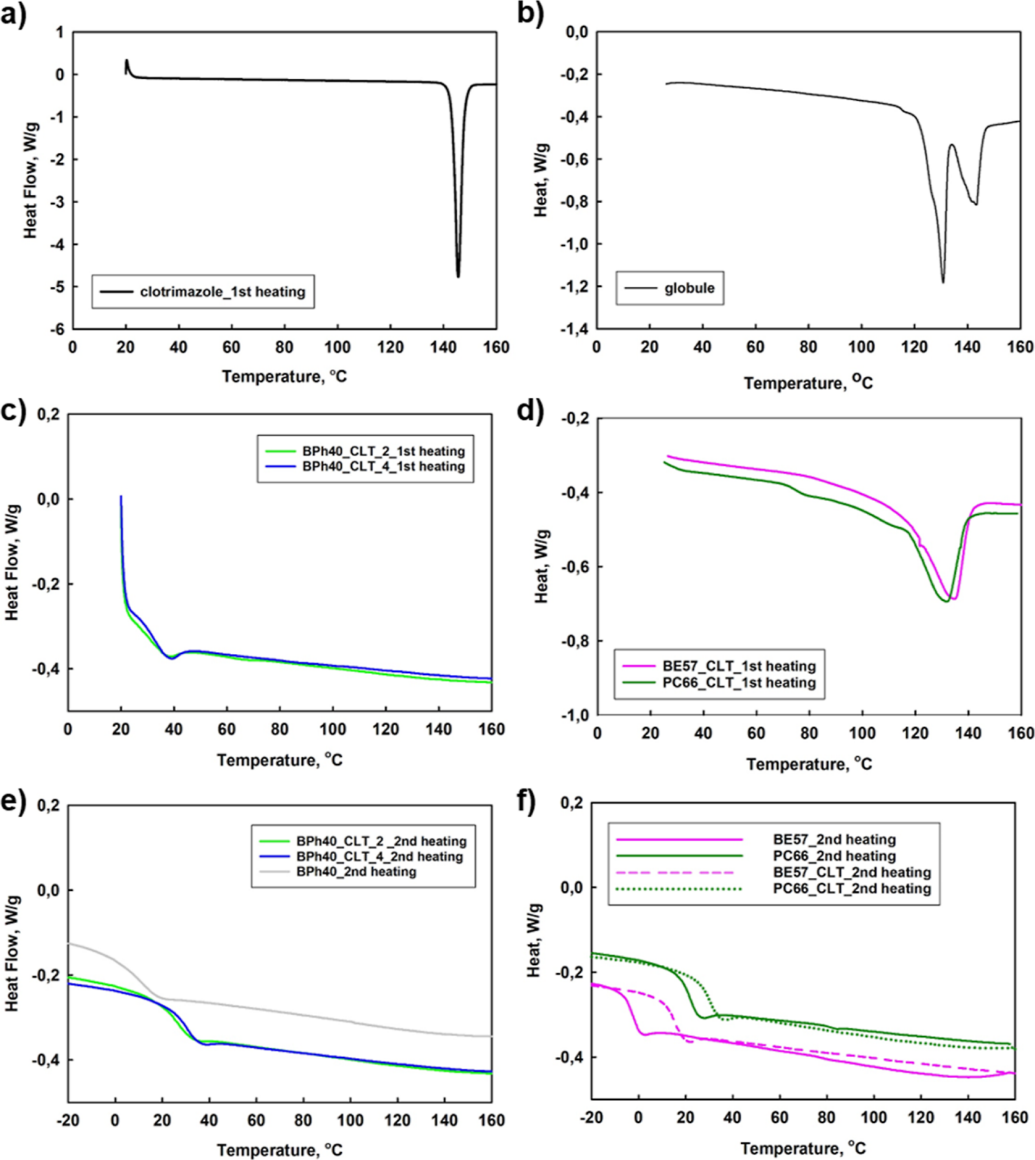
DSC thermograms at first heating recorded for neat clotrimazole (a), clotrimazole-loaded tablet (b), and clotrimazole-hydrophobized HbPGL formulations recorded at first (c,d) and at the second heating (e,f), respectively.

It is noteworthy that CLT incorporated into amphiphilic HbPGLs affected the glass transition of each of the copolymers used, which was investigated in the second heating scan (Figure 8). For example, for formulations in which the molar ratio of drug to macromolecule was 32, the *T*_g_ of BE57 and BPh40 increased from −2.8 to 15.6 °C and from 9.2 to 29.3 °C, respectively. The increase in *T*_g_ of each polymer upon CLT encapsulation is evidence of interactions between the drug and copolymer, and thus, the segmental mobility of macromolecules in the prepared formulations is markedly slowed down.

### 3.6 *In Vitro* Transdermal Permeation Study

**Figure 9:**
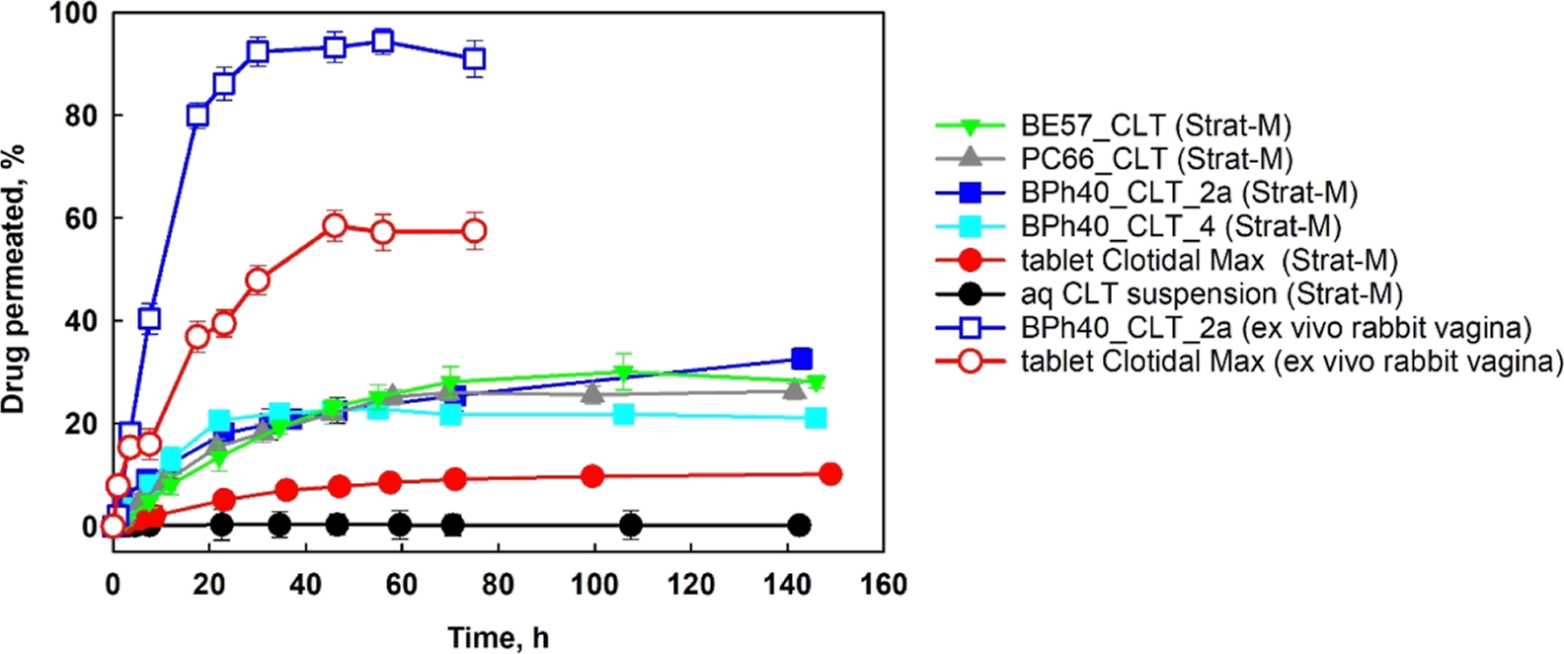
Results of a clotrimazole permeability study of various drug formulations using the Strat-M membrane or *ex vivo* rabbit vagina.

To estimate the bioavailability of CLT entrapped in the structured fluids in comparison to the commercially available CLT-loaded tablet, Clotidal Max, we performed *in vitro* permeability experiments of drug molecules from different carriers across the Strat-M membrane, *i.e.*, a synthetic nonanimal-based model for transdermal diffusion testing that is predictive of diffusion through the skin using the Franz diffusion cell. The study revealed that the level of CLT permeation through the membrane is significantly increased in the case of CLT incorporated into the matrix of aryl-enriched HbPGL-based structured fluids (Figure 9). These data indicate that hydrophobized HbPGLs are potential enhancers for the transmucosal delivery of CLT, and therefore CLT loaded in structured fluids is more bioavailable and can increase the efficiency of antifungal therapy. To quantify the impact of the drug carrier on the ability of CLT permeation through the membrane related to the permeation of CLT suspended in water or loaded in the form of commercially available formulation, we calculated the permeability constant, *K*_p_ (, where *Q* is the amount of drug transported through the membrane in time *t*, *A* is the area exposed membrane, and *C*_o_—donor concentration). *K*_p_ for CLT entrapped in structured fluids was approximately 5 times higher than it was observed for CLT loaded into a commercially available over-the-counter tablet and 50 times higher compared to CLT suspended in water (Table 4). The highest values of *K*_p_ were detected for CLT, which was loaded in the formulations based on biphenyl-modified HbPGL, *i.e.*, 3.03 × 10^–5^ and 3.35 × 10^–5^ cm/min, respectively. The flux  of CLT enclosed in the PC66-based fluid was 2.24 × 10^–4^ mg/cm^2^ min, whereas the flux of CLT from BPh40_CLT_4 was equal to 3.03 × 10^–4^ mg/cm^2^ min. The flux of CLT was the lowest from the tablet and from the aqueous suspension and was equal to 6.16 × 10^–5^ and 1.62 × 10^–6^ mg/cm^2^ min, respectively (Table 4).

**Table 4:** Comparison of the Permeability Constant (*K*_p_) of CLT from Different Formulations Permeated through an *In Vitro* STRAT-M Membrane or *Ex Vivo* Rabbit Vagina

matrix	membrane	*K*_p clotrimazole_, cm/min	*J*, mg/cm^2^ min
BE57_CLT	Strat-M	2.00 × 10^–5^	1.76 × 10^–4^
PC66_CLT	Strat-M	2.49 × 10^–5^	2.24 × 10^–4^
BPh40_CLT_2a	Strat-M	3.14 × 10^–5^	2.83 × 10^–4^
BPh40_CLT_2a	*ex vivo* rabbit vagina	1.30 × 10^–4^	1.08 × 10^–3^
BPh40_CLT_4	Strat-M	3.36 × 10^–5^	3.03 × 10^–4^
tablet (Clotidal Max)	Strat-M	6.85 × 10^–6^	6.16 × 10^–5^
tablet (Clotidal Max)	*ex vivo* rabbit vagina	5.08 × 10^–5^	4.57 × 10^–4^
aqueous suspension of clotrimazole	Strat-M	6.73 × 10^–7^	1.62 × 10^–6^

Due to the fact that formulations based on biphenyl-hydrophobized HbPGL are the most prospective for the intravaginal therapy, we additionally performed an *ex vivo* permeability test for BPh40_CLT_2a as the representative sample among biphenyl-HbPGL formulations and a Clotidal MAX tablet using excised rabbit vaginal tissue. Similarly to the permeability results obtained with the STRAT-M membrane, the formulation based on biphenyl-HbPGL assured better drug permeability through rabbit vaginal mucosa compared to a commercially available tablet. It is noteworthy that higher transmucosal transfer of CLT through the rabbit vagina was observed than through the Strat-M membrane for both drug formulations (Figure 9). The determined *K*_p_ of CLT permeated through rabbit mucosa from biphenyl-hydrophobized HbPGL-based structured fluid was approximately 3-fold higher than observed for CLT permeated from the tablet.

Permeability tests using both the artificial transdermal membrane and the *ex vivo* vagina mucosa undoubtedly show that structured fluids based on aryl-enriched HbPGLs unimolecular micelles with CLT can ensure sustained delivery of the bioactive compound to the afflicted area.

This behavior can be strictly ascribed to enhanced solubility of CLT in the aqueous medium using one polymer component for the construction of drug carriers.

### 3.7 Retention of Structured Fluids under Conditions of Simulated Vaginal Fluid

Among all investigated structured fluids constructed of hydrophobized HbPGLs, biphenyl urethane-based systems turned out to be the most perspective for intravaginal therapy in view of CLT solubility in the aqueous medium, rheological behavior, and permeability properties. Due to this fact, we investigated the retention of such constructed structured fluid in simulated vaginal fluid at 37 °C using the BPh40_CLT_2a sample, as a representative formulation. For this goal, a portion of gel-like formulation (0.03 g) was placed on a plastic surface and then immersed in 1 mL of simulated vaginal fluid. The sample was incubated for 24 h, at which the permeability plateau was attained, and the maximum CLT permeability was observed at this period of time.

**Figure 10:**
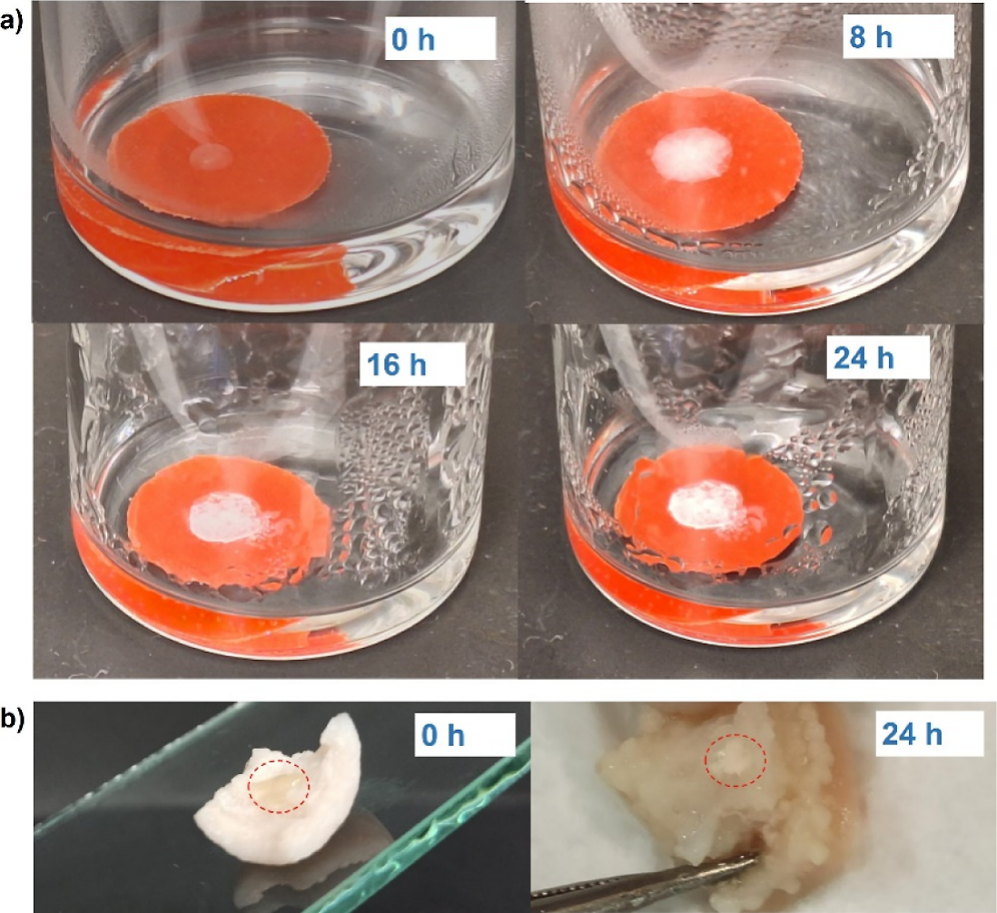
Retention of BPh40_CLT_2a formulation deposited on a plastic disc (a) and porcine skin immersed in simulated vaginal fluid at 37 °C (b).

Monitoring the sample at different time intervals allowed for the visual detection of a gradual loss of polymer from the formulation along with obtaining the opacity (Figure 10). It was detected that the coverage of the disc with a sample doubled in several hours. However, the drug formulation persisted as a continuous film on the surface. ^1^H NMR analysis of the dried residue after 24 h of incubation in SVF showed that it consisted of both drug and polymer. The same behavior was observed for the formulation deposited on the porcine skin.

Due to the fact that *in vivo* vaginal mucosa undergoes recurring cycles of proliferation at the basal layer, maturation, and desquamation into the vaginal lumen, with a turnover time of about 96 h,^38^ the drug-formulation based on structured fluids based on hydrophobized HbPGLs should be completely removable.

### 3.8 *In Vitro* Antifungal Activity

**Figure 11:**
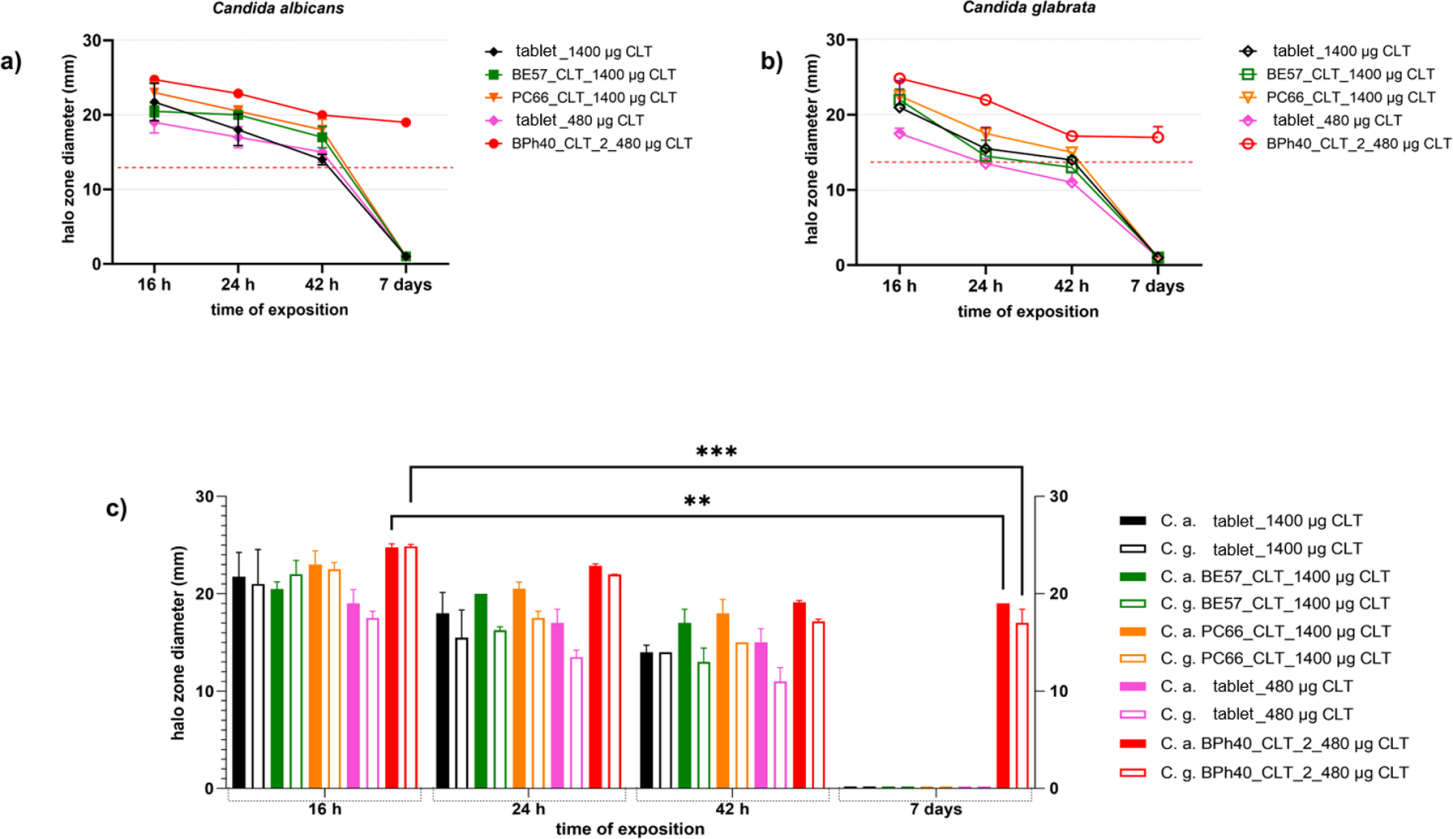
Halo zone diameters determined for clotrimazole-loaded in the tablet and clotrimazole formulations with aryl-modified HbPGLs against *C.albicans* (a) and *C. glabrata* (b) and comparison of anti-*C. albicans* and *C. glabrata* activity of clotrimazole formulations (c).

To estimate the antifungal potential of the synthesized structured fluids loaded with CLT, we compared their antifungal properties with the activity of over the counter commercially available CLT-loaded tablet, Clotidal MAX. The antifungal activity was validated by the presence of the zone of inhibition against *C. albicans* and *C. glabrata*. Based on the values of the halo zone diameter (Figure 11), it was demonstrated that the antifungal activity of CLT loaded into constructs BE57 and PC66 against both *C. albicans* and *C. glabrata* is comparable to the activity of a commercially available CLT-loaded tablet for the treatment of vaginal and vulvar fungal infections. The halo zone for all constructs and discs with controls remained statistically unchanged for up to 42 h. In the later period, both *C. albicans* and *C. glabrata* showed intensive growth around the discs saturated with constructs BE57_CLT, PC66_CLT containing 1400 μg of CLT, as well as with the commercially used CLT in the tablet in the amount equal to 480 μg as well as 1400 μg. Although at almost every measurement time point, the diameter of the halo zone was higher for *C. albicans*, no statistical significance was observed in relation to the values of the growth inhibition zones obtained for *C. glabrata*. Surprisingly high antifungal activity was observed against both *Candida* strains in the case of construct BPh40_CLT_2a, containing 65% less loaded CLT in comparison to phenyl-enriched HbPGLs, *i.e.*, the BE57_CLT and PC66_CLT formulations. Furthermore, it was demonstrated that the growth inhibition zones persisted until the seventh day of the conducted study, which was terminated due to the loss of nutritional properties of the growth medium itself (Sabouraud agar). In each time measurement point, *i.e.*, 16, 24, and 42 h, and 7 days, construct BPh40_CLT_2a exhibited the highest activity against both reference strains of *Candida* used in the experiment. Furthermore, this construct exhibited the highest growth inhibition zone (compared to BE57_CLT and PC66_CLT and the CLT control at both *c* = 480 μg and *c* = 1400 μg), reaching a value of nearly 25 mm after 16 h. A statistically significant (for *C. albicansp* = 0.01 and *C. glabratap* = 0.001) growth increase (22% for *C. albicans* and 32% for *C. glabrata*) around the discs saturated with the constructs BPh40_CLT_2a (approximately: 5.5 mm and 8 mm accordingly) was observed at 42 h in compared to the measurement from the initial time point and remained at an unchanged level until the seventh day of the conducted observation. Based on the obtained results, the construct BPh40_CLT_2a containing CLT at *c* = 480 μg was selected as a promising agent against *C. albicans* and *C. glabrata*, maintaining a 7 day activity against fungal used strains, which is highly significant in disease management, particularly in female genital tract infections.

## 4 Conclusions

In this paper, we have elaborated the method of efficient CLT solubilization in water in the form of structured fluids using HbPGLs of a hydrophobized core with aryl moieties, such as benzoyl ester, phenyl urethane, and biphenyl urethane. The properties of structured fluids were strictly dependent on the type of aryl-HbPGL derivative used. The biphenyl-HbPGL derivative showed the most significantly enhanced CLT solubilization in the aqueous media among the investigated amphiphilic constructs. All CLT formulations constructed of biphenyl-HbPGL derivatives, independently of drug to polymer molar ratio varying from 8 to 32, provided transparent, molecularly dissolved formulations, which was confirmed with FTIR and DSC investigations. In the case of phenyl-based HbPGL derivatives, a minor fraction of the drug was only molecularly dispersed, and most of its amount was in crystalline form. In addition, aqueous formulations of CLT with biphenyl-HbPGL derivatives showed more favorable rheological properties in contrast to those of phenyl-HbPGL derivatives and were more stable under storage conditions. Depending on the molar ratio of the drug to the polymer, the formulation exhibited a yield limit at body temperature or a viscosity that ensured film formation on the vaginal tissue, and therefore, the flow of the drug carrier was limited. Thus, these formulations ensure prolonged action of the active substance with the afflicted site. The percutaneous permeability of CLT loaded in aryl-enriched HbPGLs was significantly increased compared to CLT from its aqueous suspension and CLT present in commercially available tablets, but with no apparent difference between aryl-HbPGL-based systems. The antifungal test showed increased activity of the aqueous biphenyl-HbPGL formulation against *C. albicans* and *C. glabrata* species, but also prolonged activity for up to 7 days of activity with a single dose of the formulation. In this work, we demonstrated a first aqueous formulation requiring the usage of a single polymer component to attain both high drug solubility and rheological properties suitable for intravaginal applications.

## Supporting Information Available

^1^H and ^13^C NMR spectra of aryl-enriched HbPGLs and intermediate products, additional rheological results, FTIR spectra of aryl-enriched HbPGLs, CLT in both crystalline and in solubilized forms (in methanolic solution), and CLT-loaded in amphiphilic polymers (PDF)
