# Supplementary material for: Enhanced Solubility and Bioavailability of Clotrimazole in Aqueous Solutions with Hydrophobized Hyperbranched Polyglycidol for Improved Antifungal Activity
Source: ACS Appl Mater Interfaces. 2024 Apr 5;16(15):18434–48. doi: 10.1021/acsami.3c19388 (PMC11040572; doi:10.1021/acsami.3c19388)
Supplement: Supplementary file 1 — am3c19388_si_001.pdf [file am3c19388_si_001.pdf]

Supporting Information

Enhanced solubility and bioavailability of clotrimazole in aqueous solutions with hydrophobized hyperbranched polyglycidol for improved antifungal activity

Daria Jaworska-Krych[a], Monika Gosecka[a]*, Mateusz Gosecki[a], Malgorzata Urbaniak[a], Katarzyna Dzitko[b], Anita Ciesielska[b], , Ewelina Wielgus[a], Slawomir Kadlubowski[c] Marcin Kozanecki[d]

a. Centre of Molecular and Macromolecular Studies, Polish Academy of Sciences, Sienkiewicza 112, 90-363 Lodz, Poland

b. University of Lodz, Department of Molecular Microbiology, Faculty of Biology and Environmental Protection, Banacha 12/16, 90-237, Lodz, Poland

c. Lodz University of Technology, Institute of Applied Radiation Chemistry, Wroblewskiego 15 93-590 Lodz, Poland

d. Department of Molecular Physics, Faculty of Chemistry, Lodz University of Technology, Zeromskiego 116, 90-924 Lodz, Poland

Corresponding Author: Monika Gosecka* - Email: mdybko@cbmm.lodz.pl

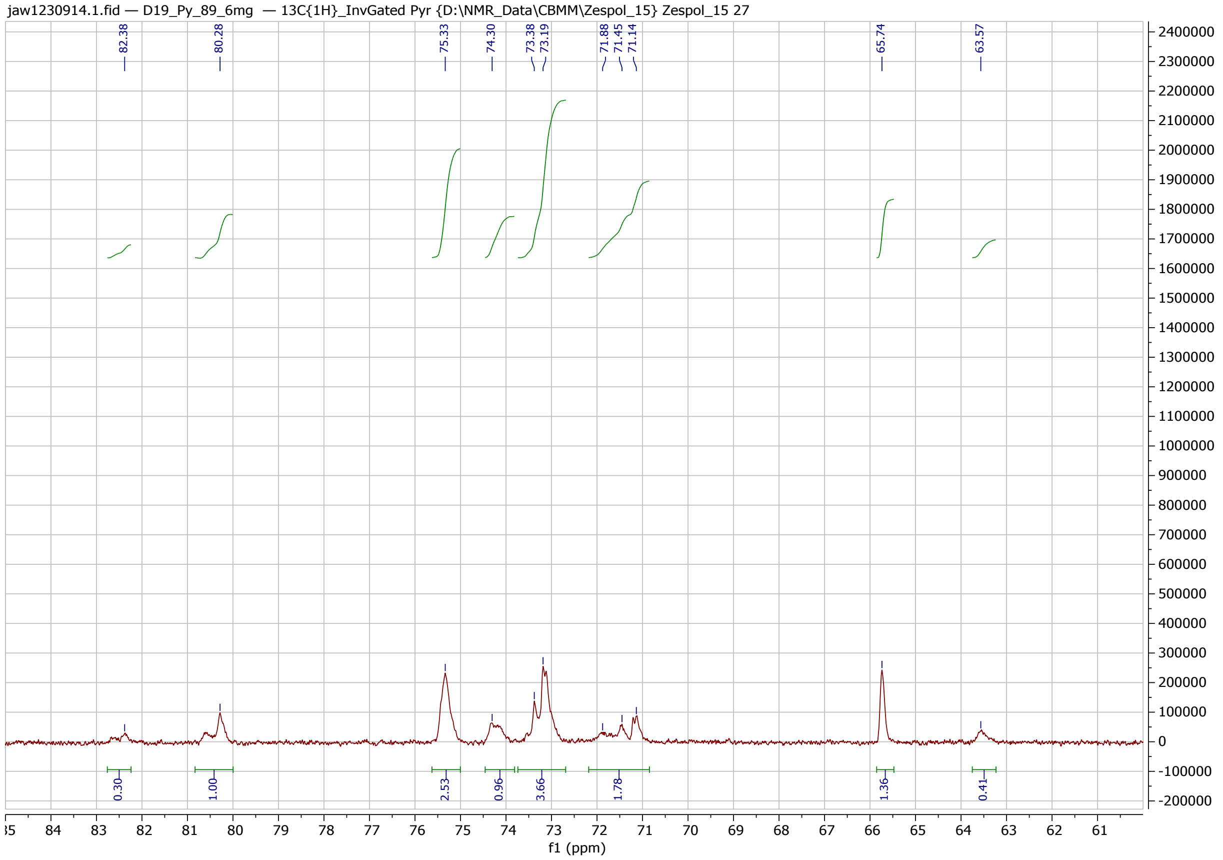

Figure S1. ^{13}C INVGATED NMR spectrum of HbPGL recorded in Pyridine-d$_5$.

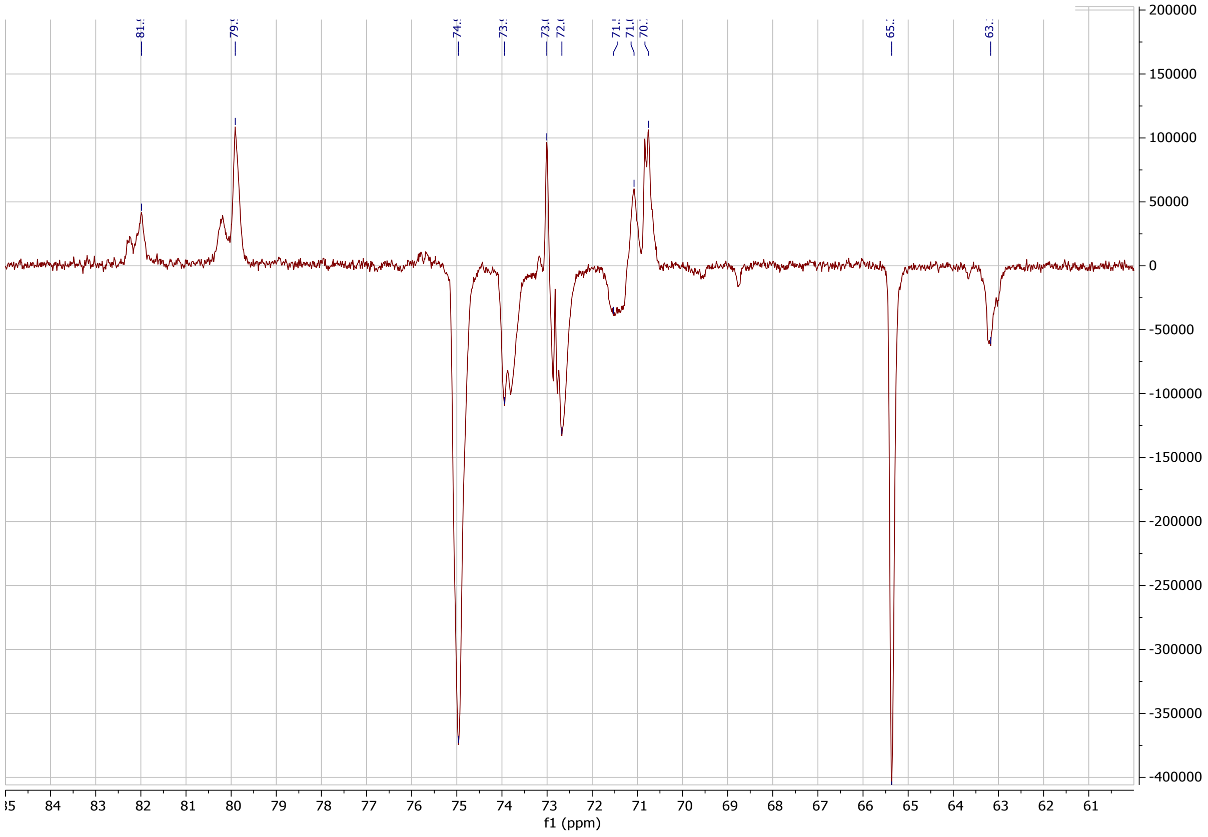

Figure S2. ^{13}C DEPT NMR spectrum of HbPGL recorded in Pyridine-d$_5$.

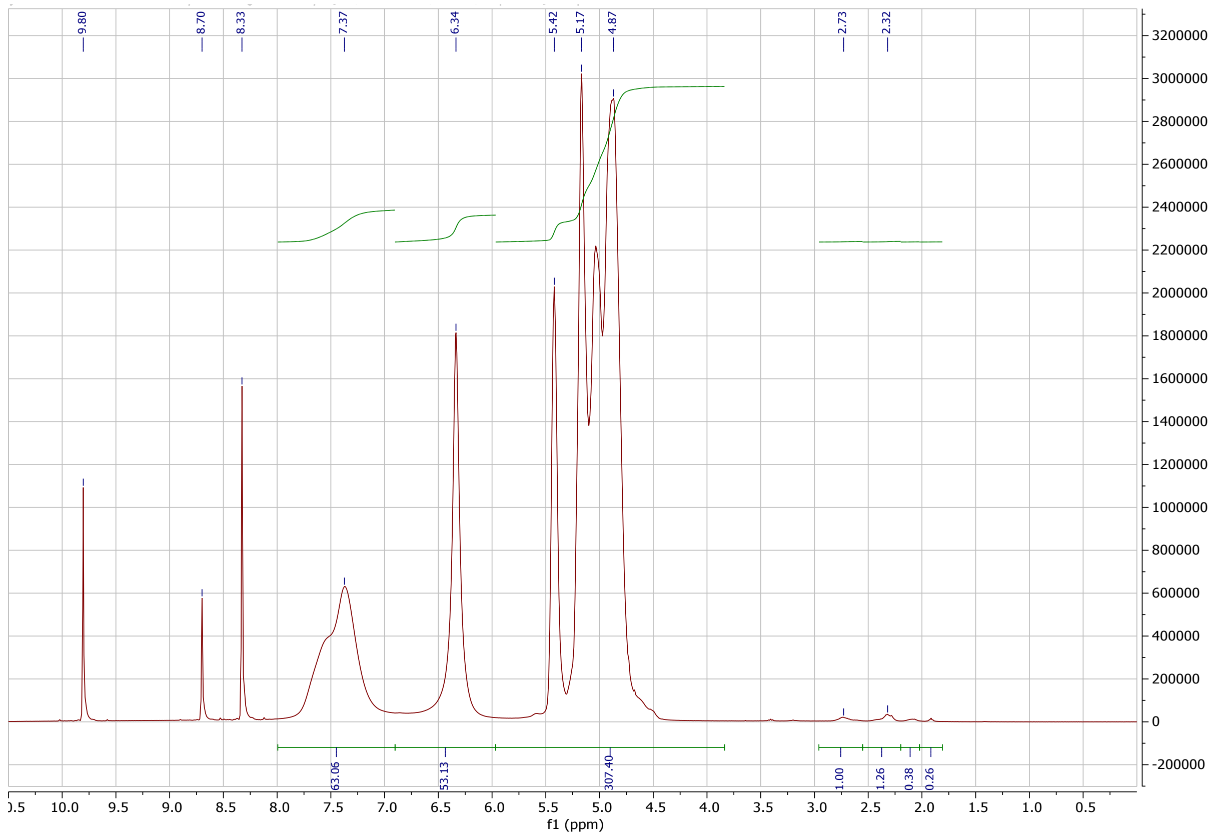

Figure S3. [1] H NMR spectrum of HbPGL recorded in Pyridine-d_5.

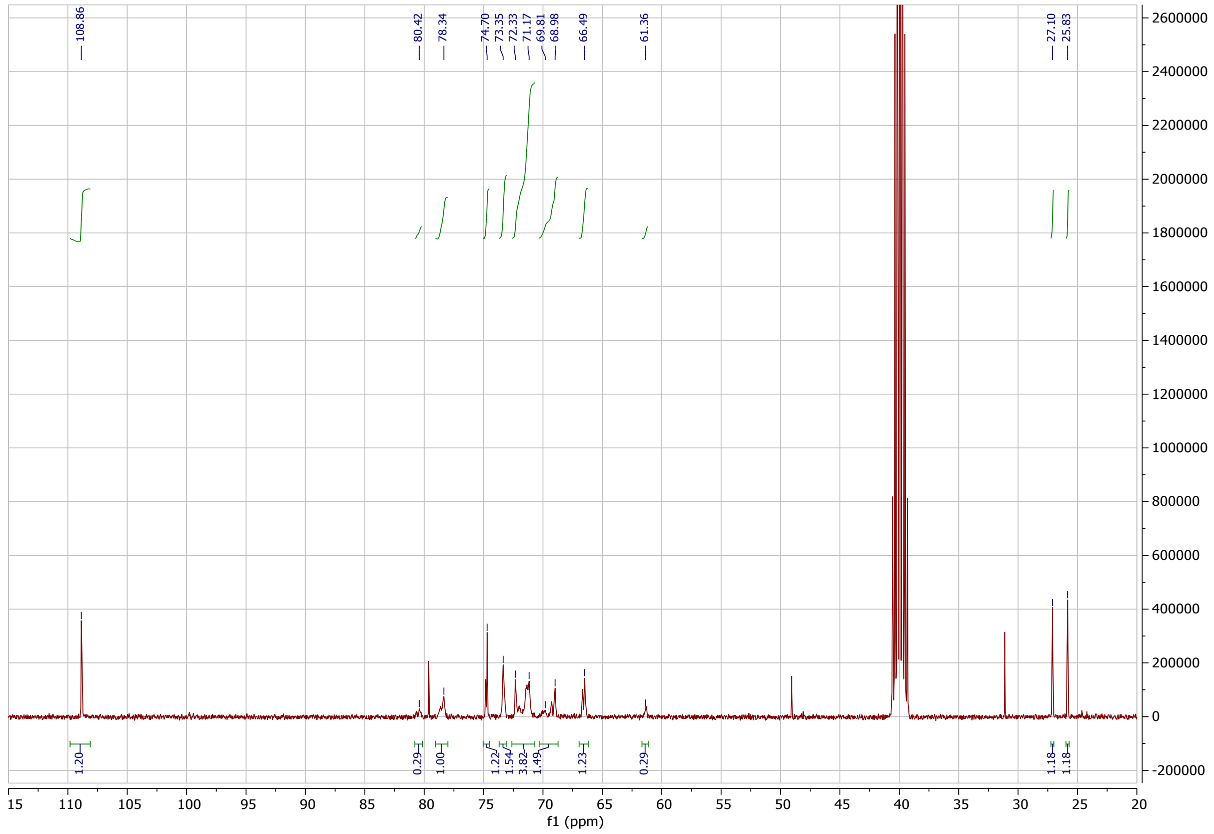

Figure S4. ^{13}C INVGATED NMR spectrum of AC-HbPGL recorded in DMSO-d_6.

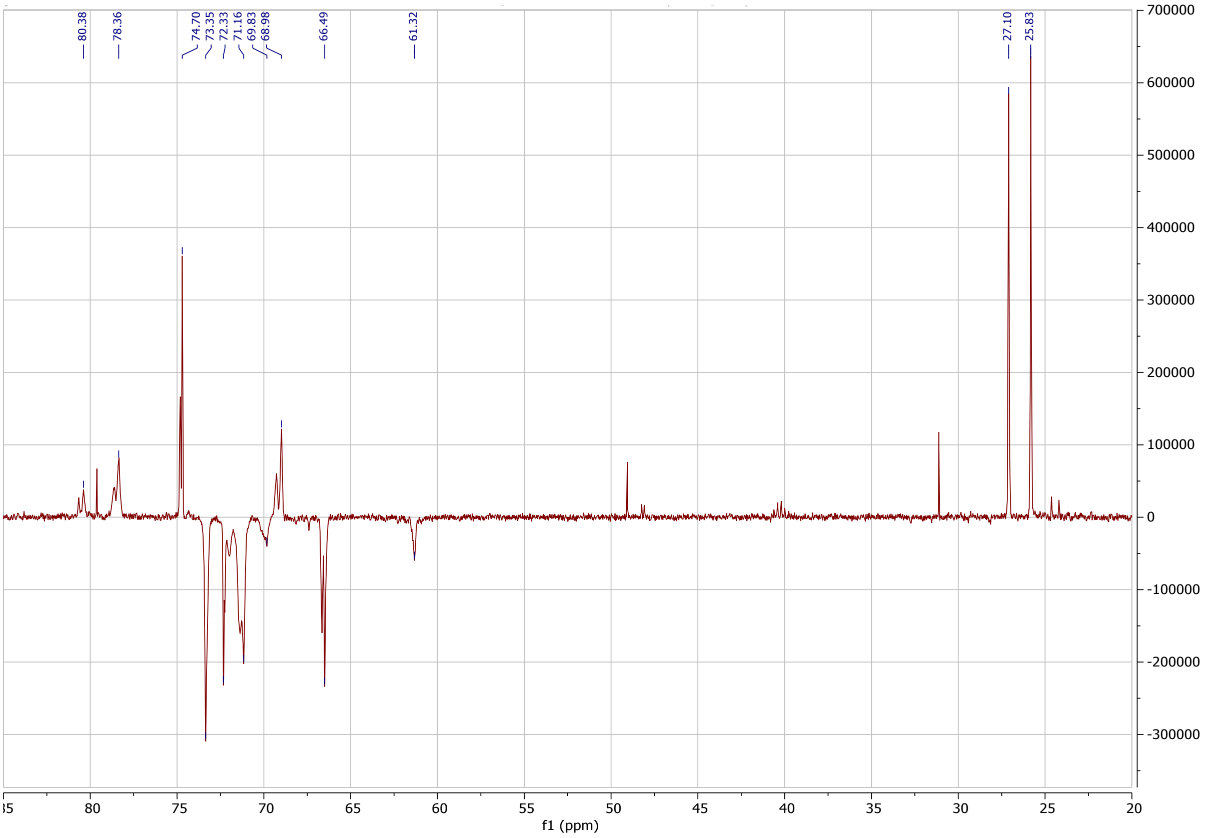

Figure S5. ^{13}C DEPT NMR spectrum of AC-HbPGL recorded in DMSO-d$_6$.

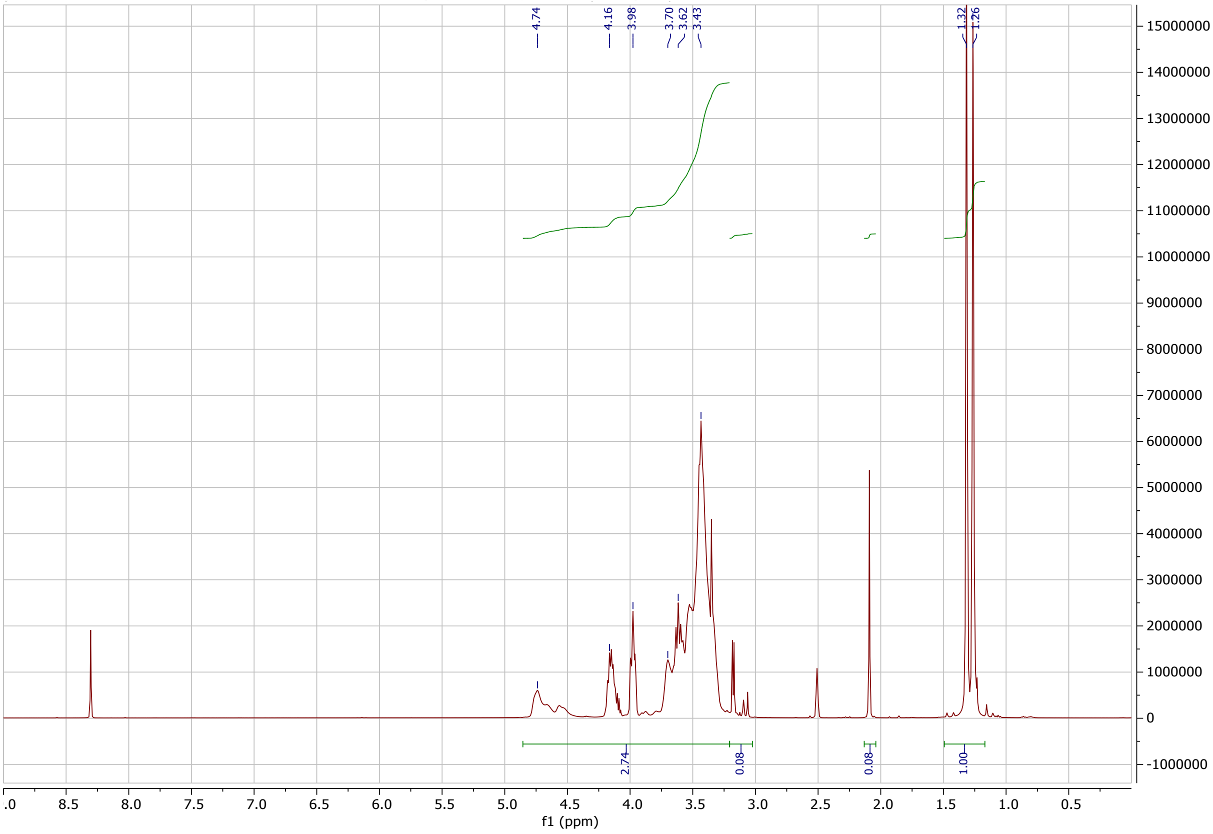

Figure S6. ^{1}H NMR spectrum of AC-HbPGL recorded in DMSO-d$_6$.

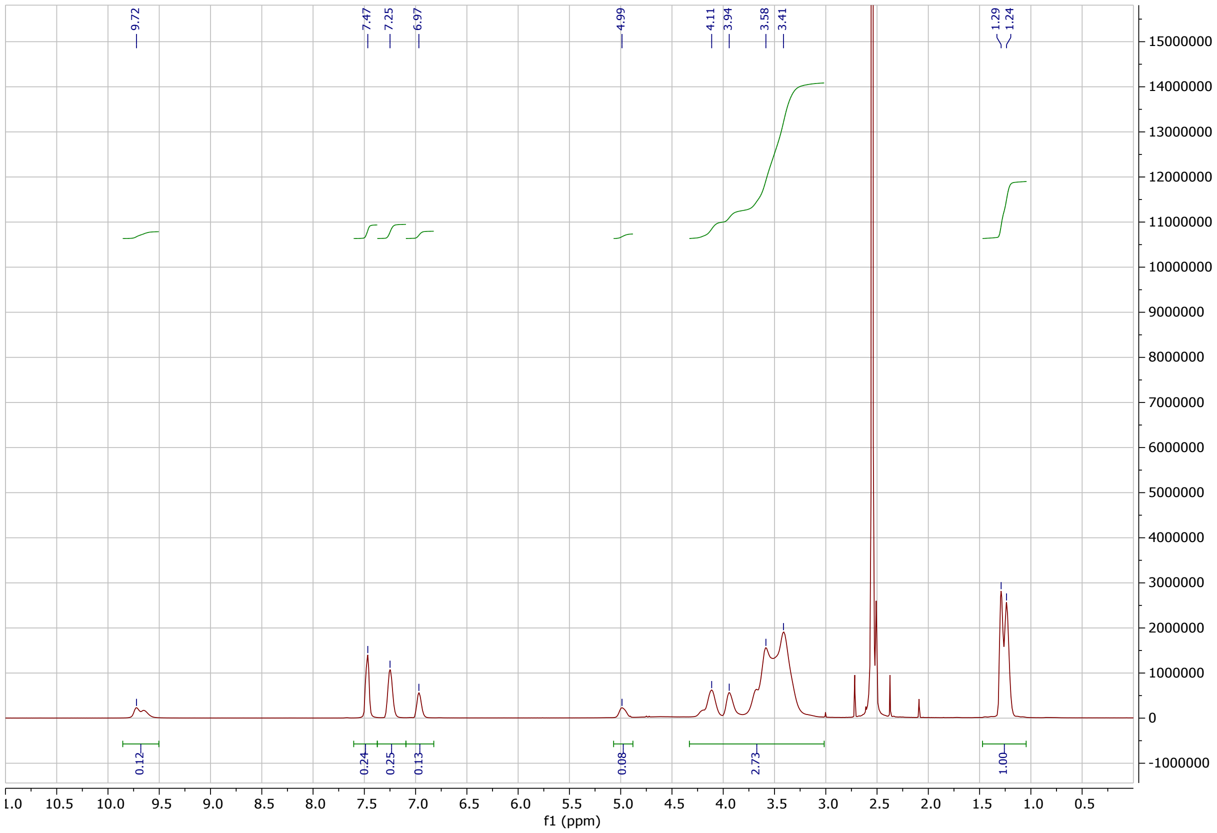

Figure S7. ^{1}H NMR spectrum of AC-HbPGL_PC66 recorded in DMSO-d_6.

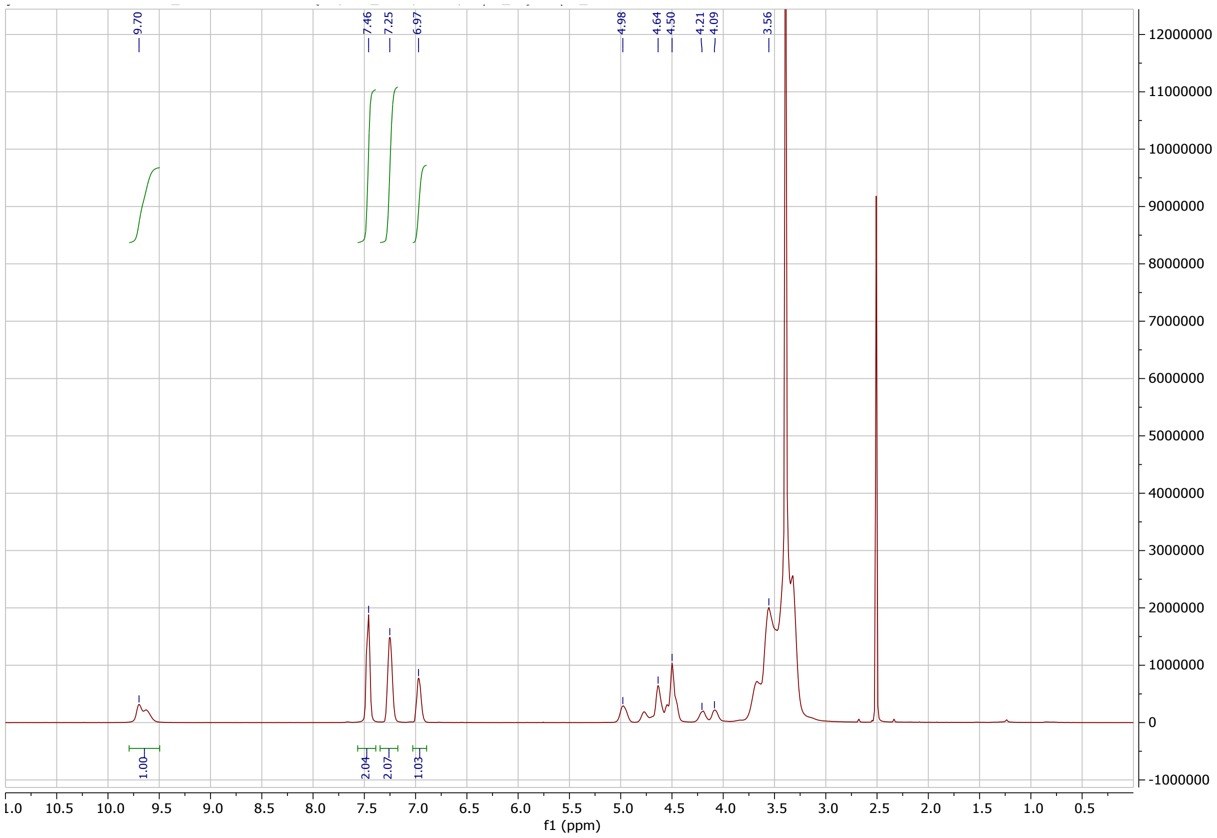

Figure S8. ^{1}H NMR spectrum of HbPGL_PC66 recorded in DMSO-d_6.

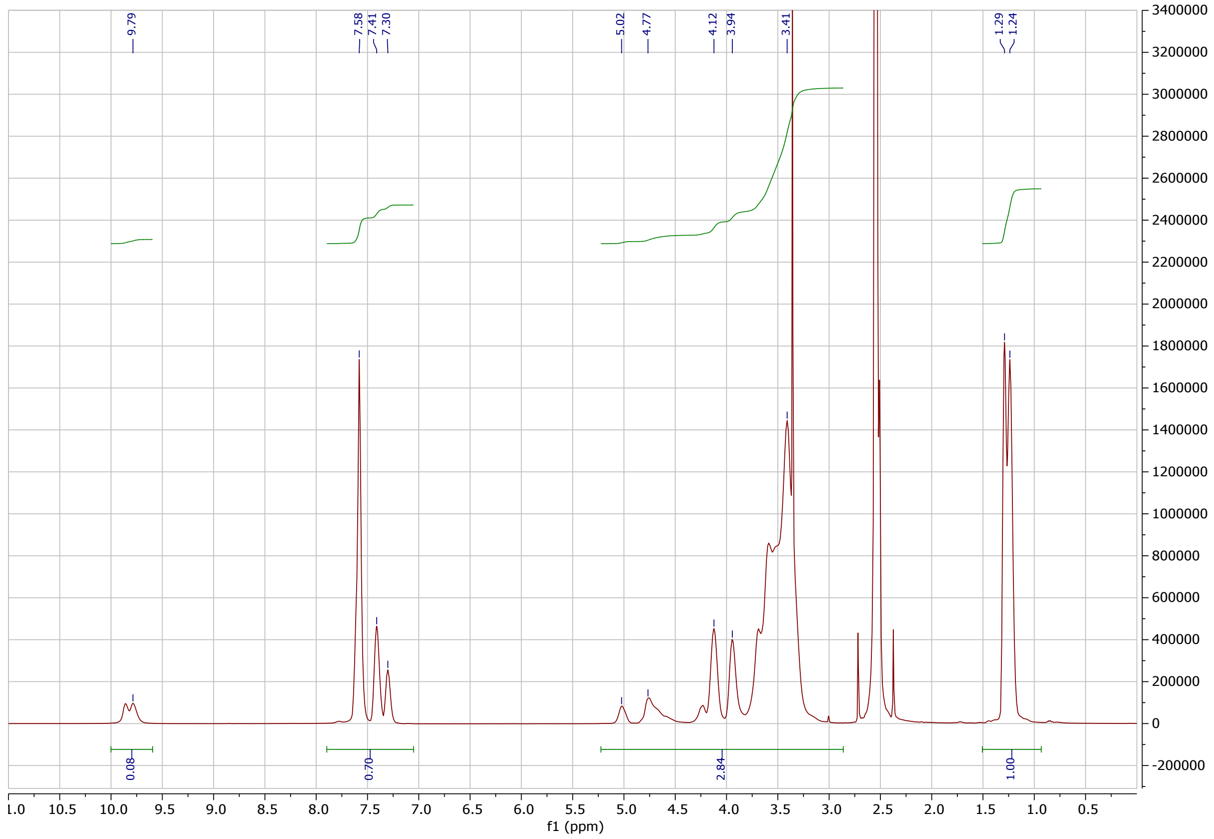

Figure S9. ^{1}H NMR spectrum of AC-HbPGL_BPh40 recorded in DMSO-d_6.

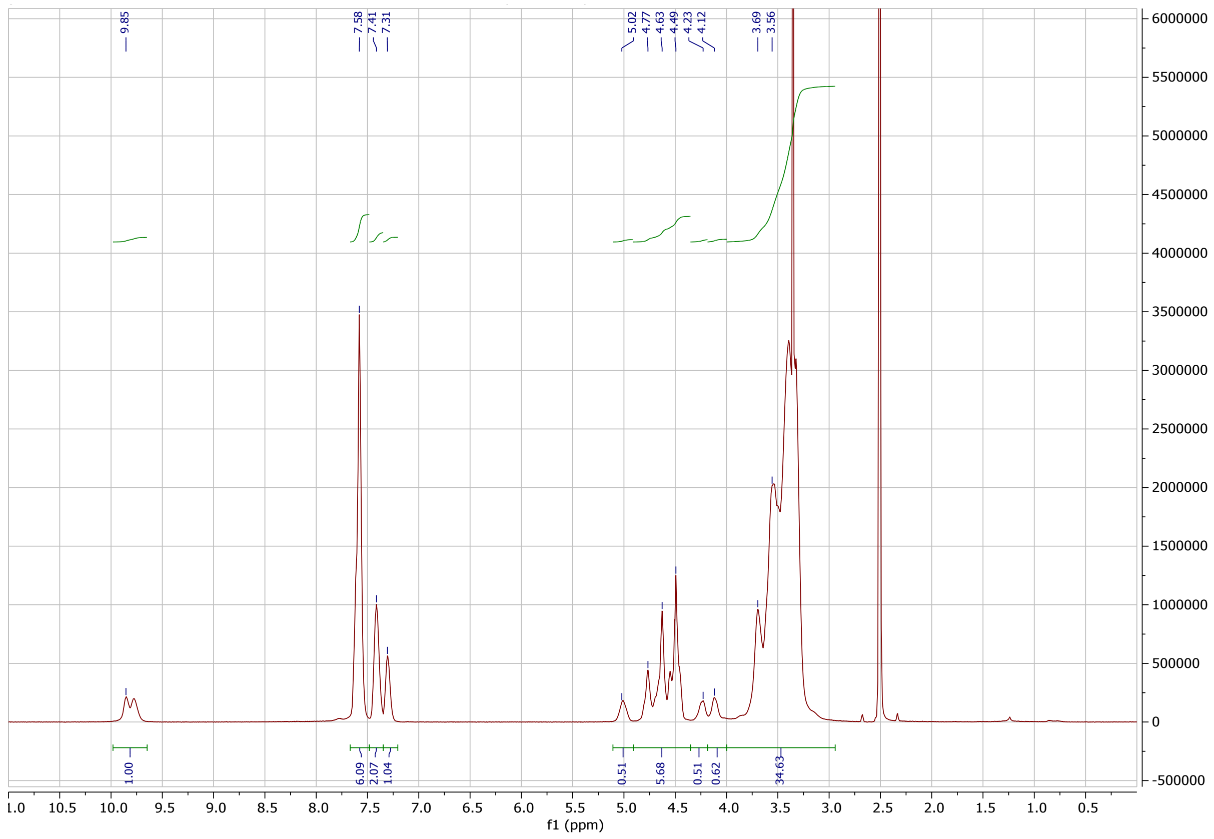

Figure S10. ^{1}H NMR spectrum of HbPGL_BPh40 recorded in DMSO-d_6.

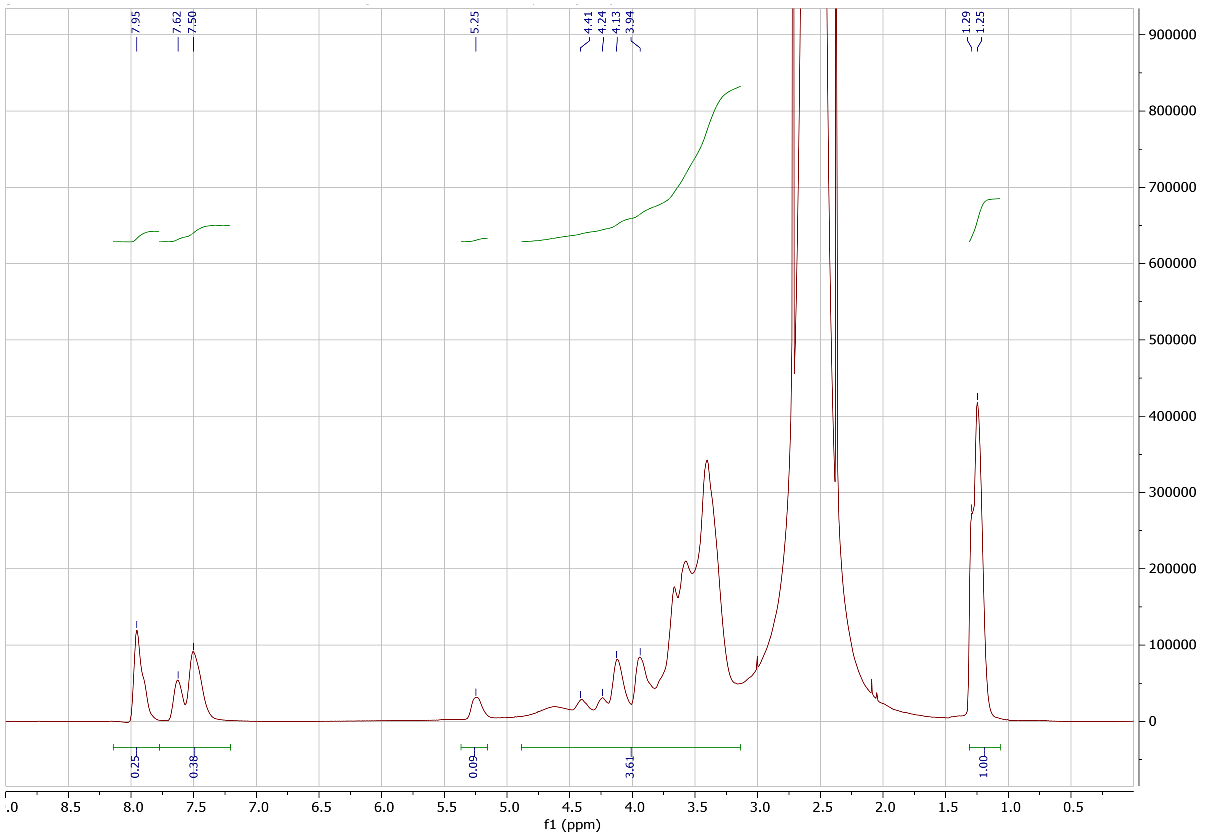

Figure S11. ^{1}H NMR spectrum of AC-HbPGL_BE57 recorded in DMSO-d$_6$.

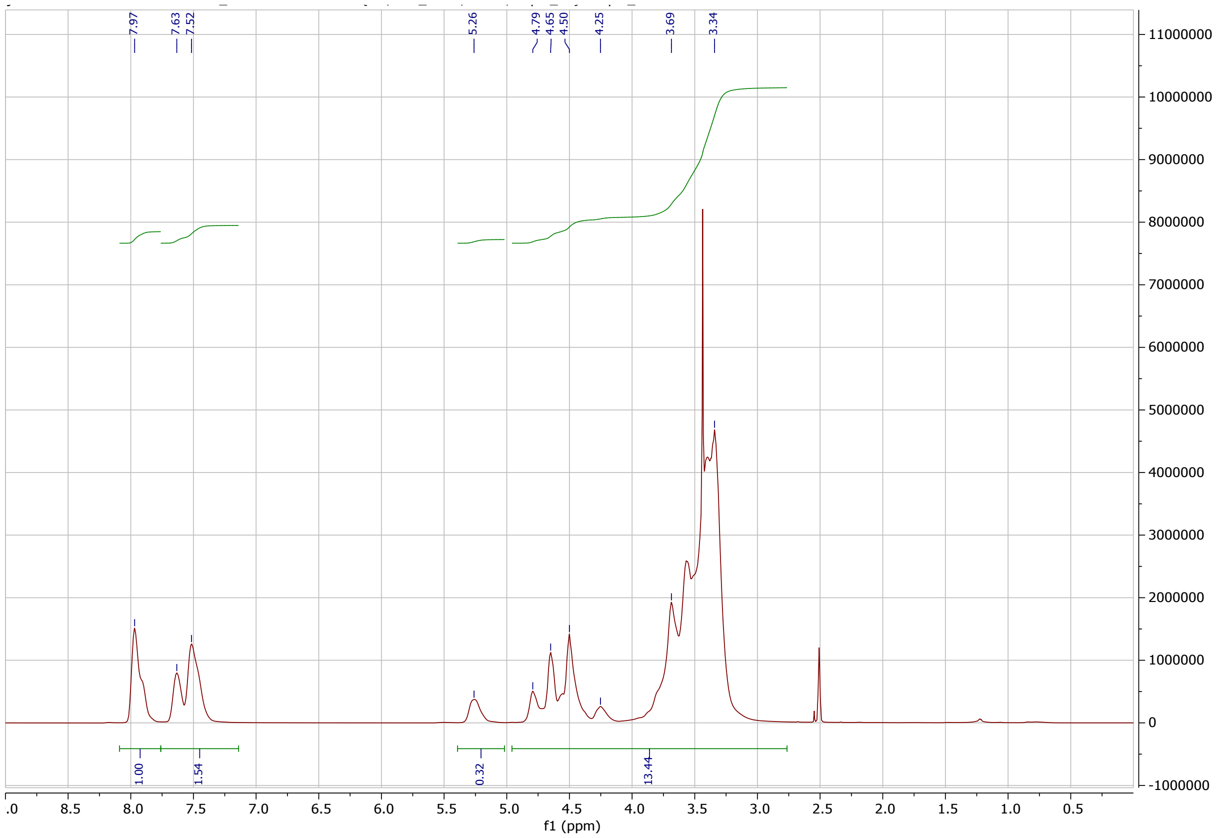

Figure S12. ^{1}H NMR spectrum of HbPGL_BE57 recorded in DMSO-d$_6$.

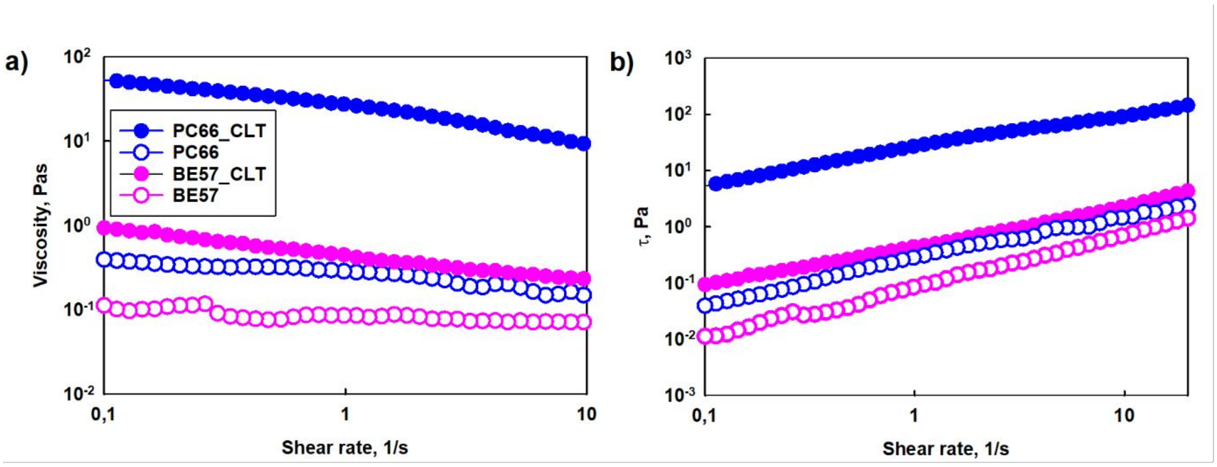

Figure S13. The dependence of viscosity (a) and stress (b) on the shear rate recorded at 37 ^{o}C for the aqueous solutions of phenyl-based HbPGLs and aqueous formulations prepared of clotrimazole-loaded phenyl-based HbPGLs.

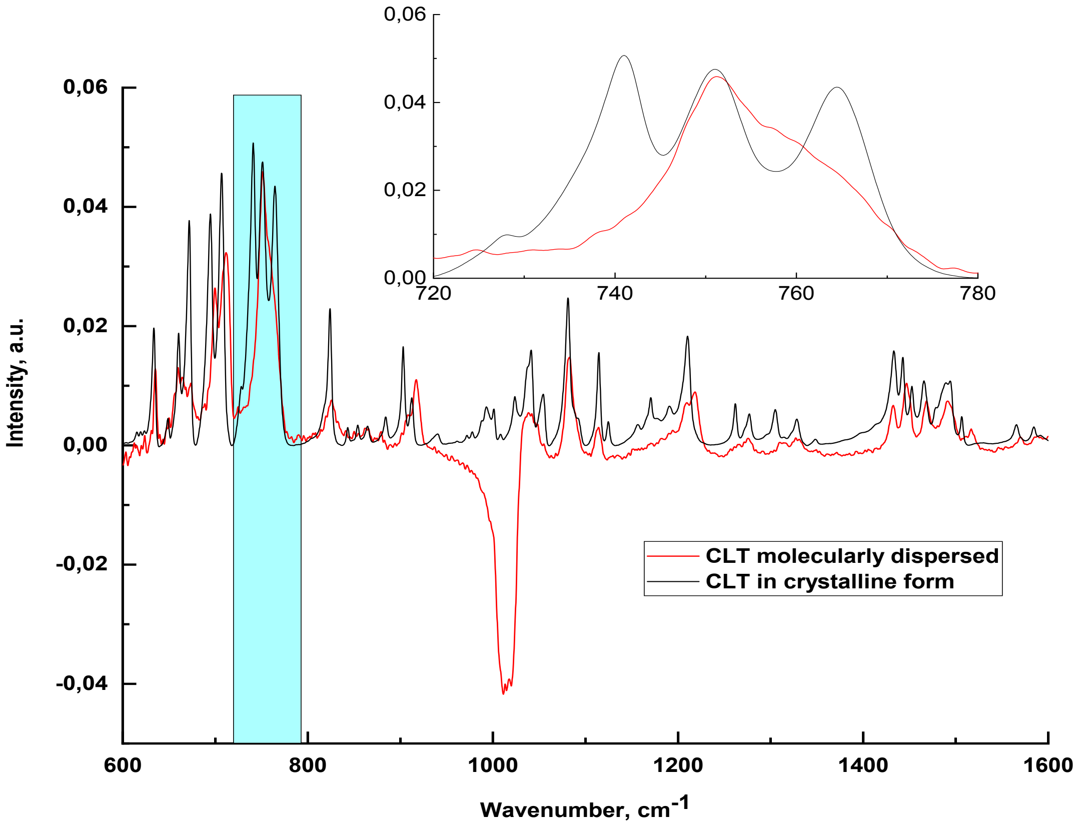

Figure S14. The comparison of FTIR spectrum of clotrimazole in crystalline form and differential spectrum of the FTIR spectra of methanolic solution of clotrimazole and methanol. Inset shows the spectral region 720-780 cm^{-1} sensitive to clotrimazole crystallinity.

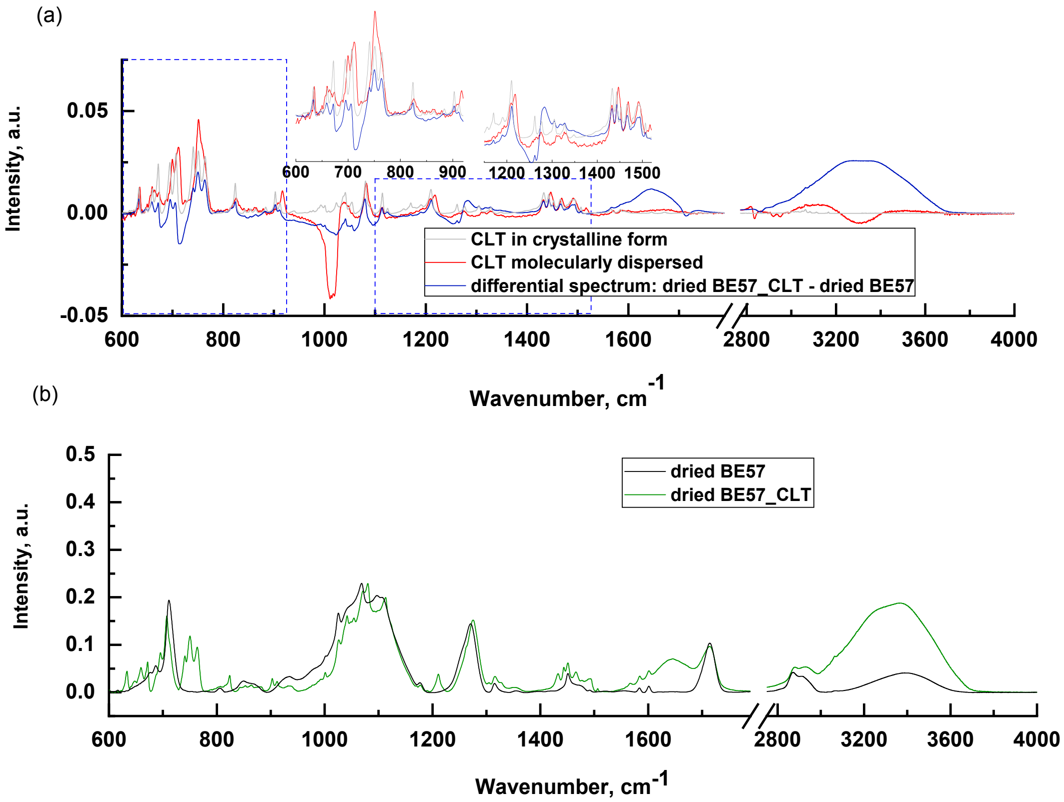

Figure S15. The comparison of FTIR spectra of clotrimazole in crystalline form, a differential spectrum of the FTIR spectra of methanolic solution of clotrimazole and methanol, and clotrimazole encapsulated in polymer (a). FTIR spectra of dried neat BE57 and BE57 loaded with clotrimazole (b).

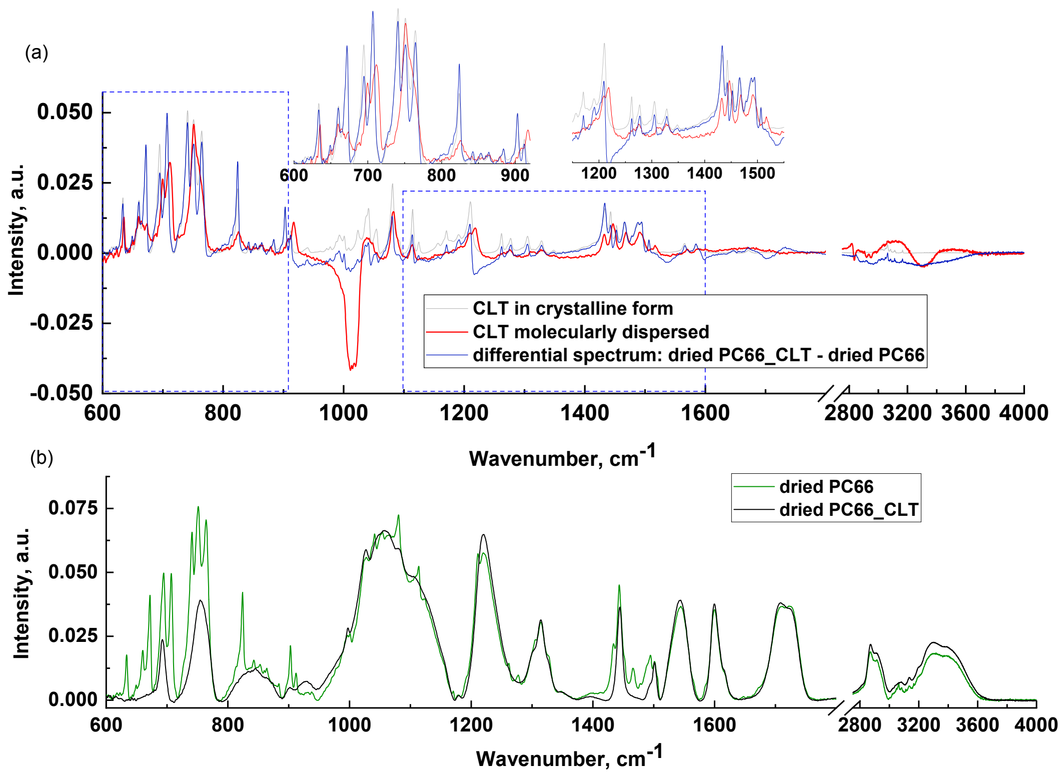

Figure S16. The comparison of FTIR spectra of clotrimazole in crystalline form, differential spectrum of the FTIR spectra of methanolic solution of clotrimazole and methanol and clotrimazole encapsulated in polymer (a). FTIR spectra of dried neat PC66 and PC66 loaded with clotrimazole (b).

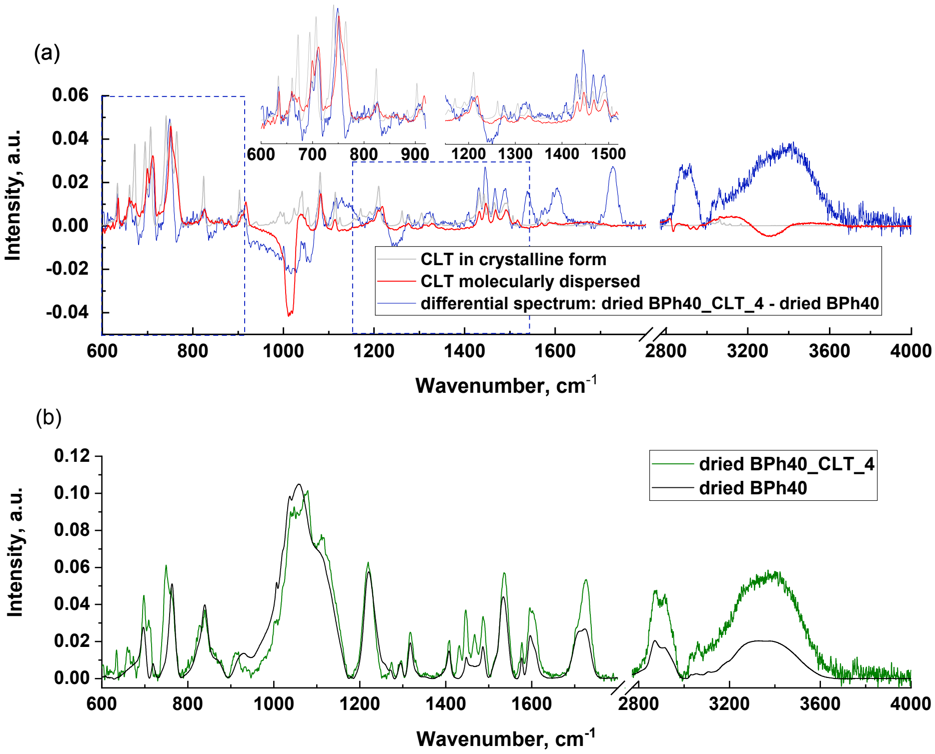

Figure S17. The comparison of FTIR spectra of clotrimazole in crystalline form, differential spectrum of the FTIR spectra of methanolic solution of clotrimazole and methanol and clotrimazole encapsulated in polymer (a). FTIR spectra of dried neat BPh40 and BPh40 loaded with clotrimazole in the molar ratio equal to 32 (b).

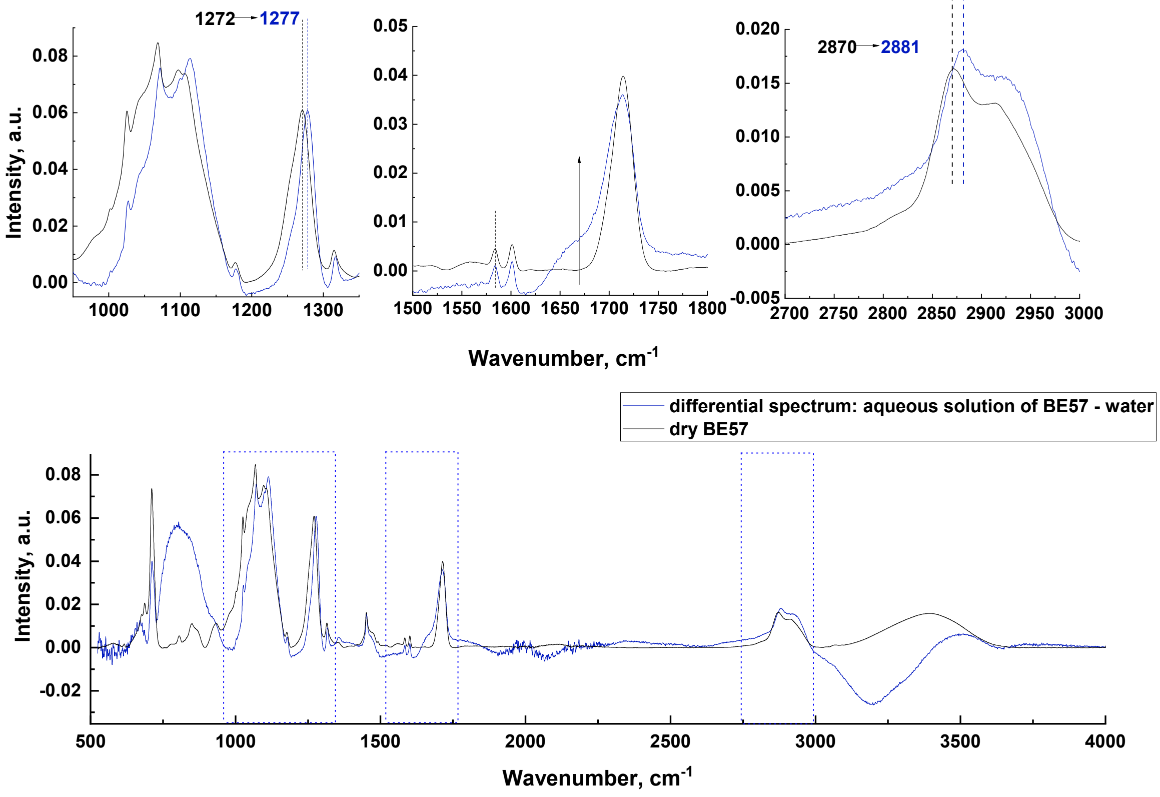

Figure S18. FTIR spectrum of BE57 in dry and hydrated states. The insets show the spectral regions where the bands of the BE57 sensitive to hydration occur.

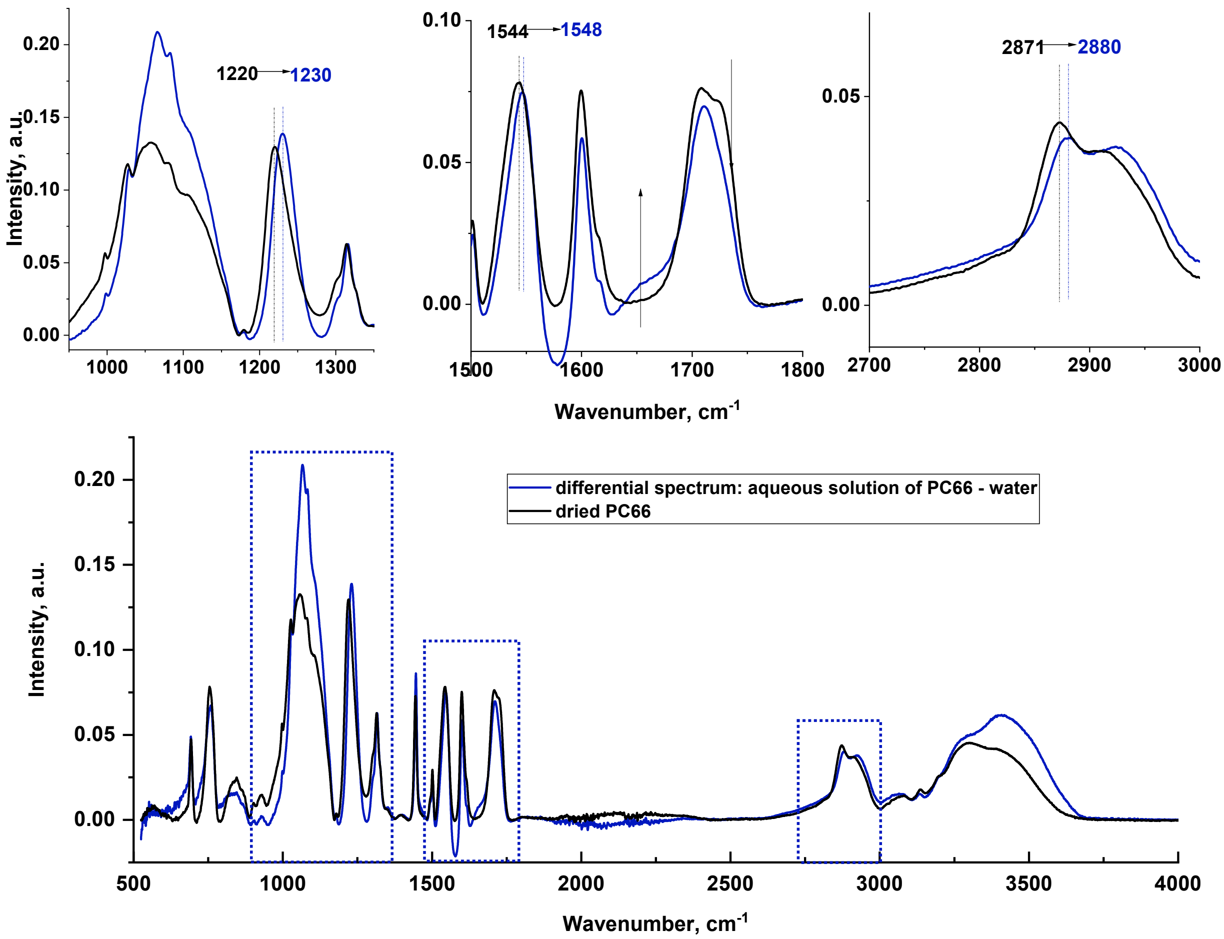

Figure S19. FTIR spectra of PC66 in dried and hydrated states. Insets show the spectral regions where the bands of the PC66 polymer are sensitive to hydration occur.

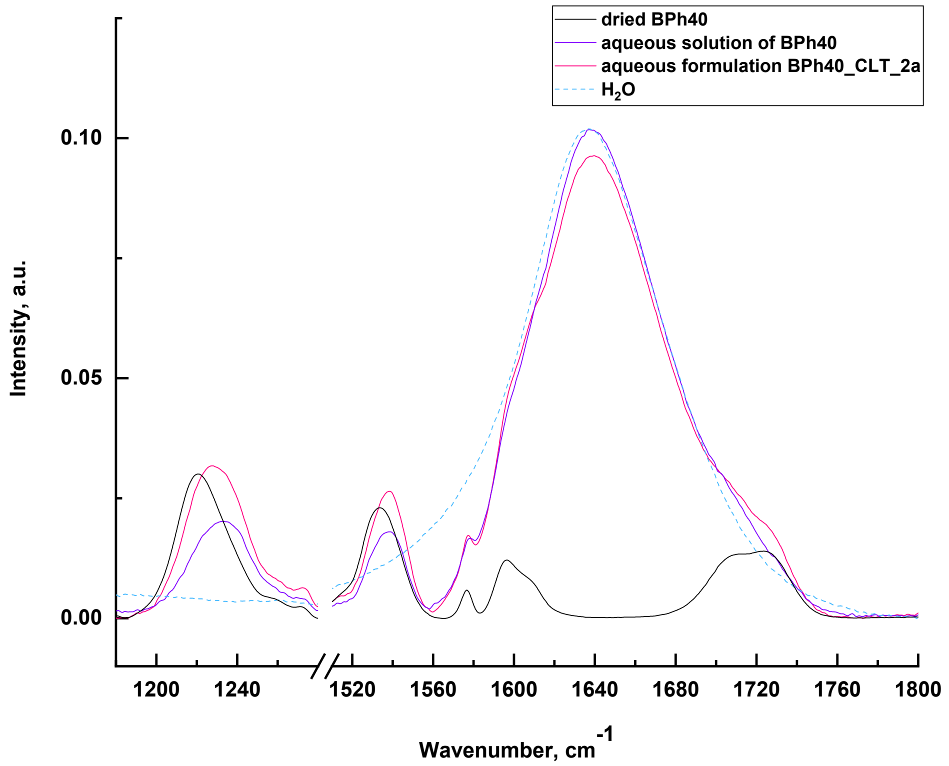

Figure S20. Comparison of FTIR spectra of dried neat BPh40, aqueous solution of BPh40, and aqueous formulation of BPh40 loaded with clotrimazole and water.

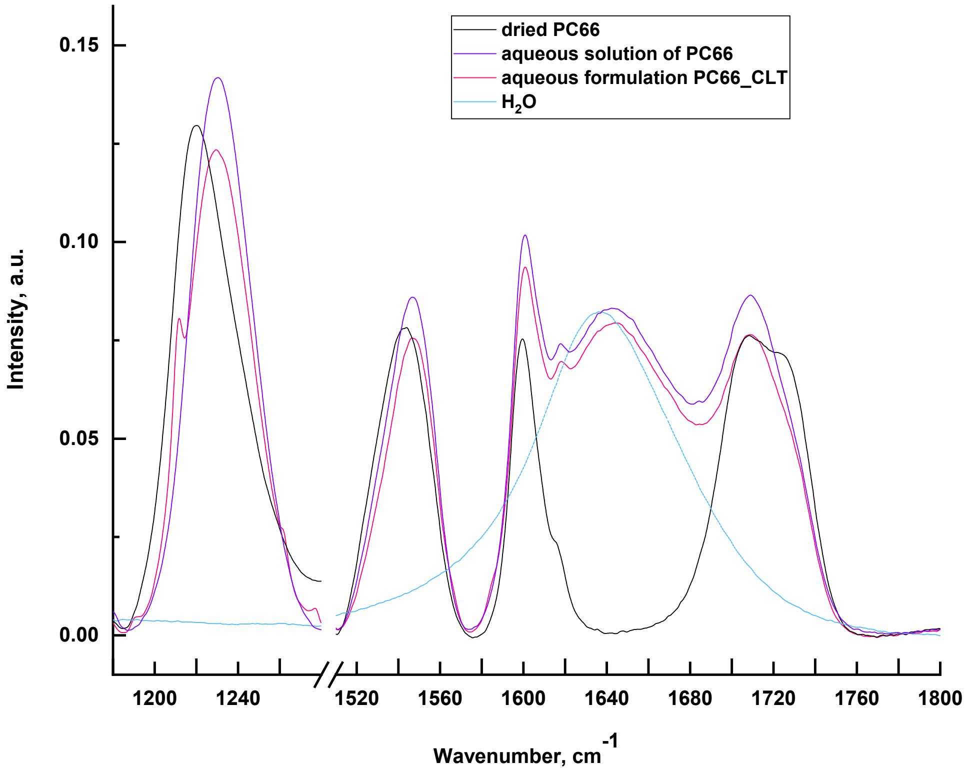

Figure S21. Comparison of FTIR spectra of dried neat PC66, aqueous solution of PC66, aqueous formulation of PC66 loaded with clotrimazole and water.

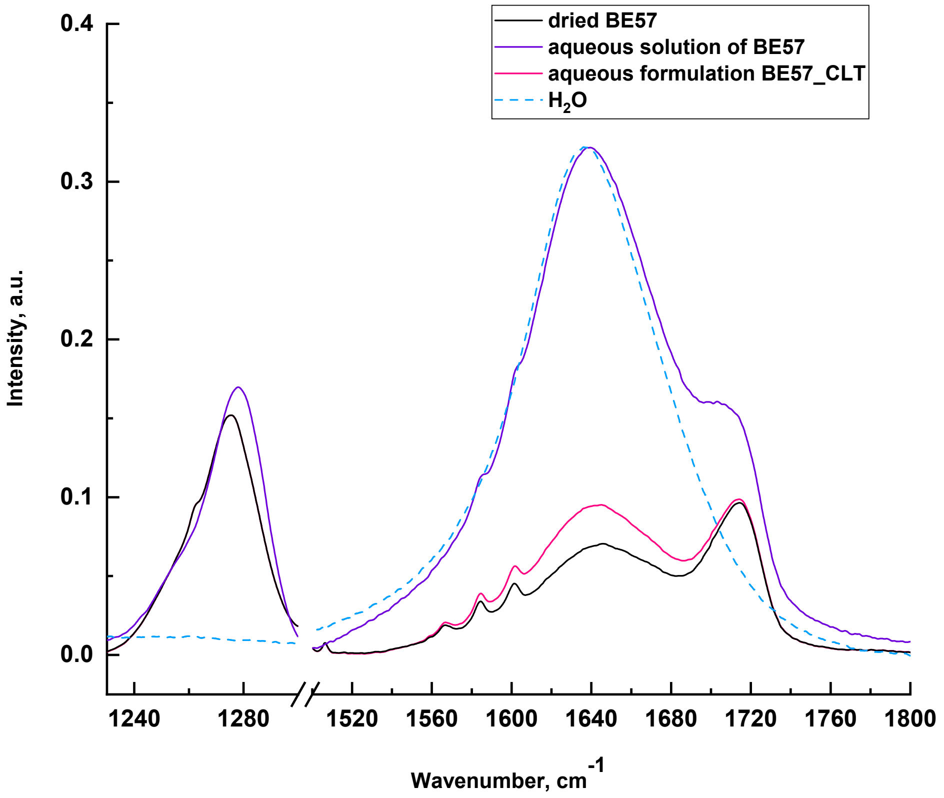

Figure S22. Comparison of the FTIR spectra of dried neat BE57, aqueous solution of BE57, aqueous formulation of BE57 loaded with clotrimazole and water.

Table 1. Zero viscosity values (η_0) of formulations based on clotrimazole-loaded aryl-modified hyperbranched polyglycidols with the indication of a presence of the yield point (τ_c).

**Aqueous formulation**	**Molar ratio of clotrimazole to copolymer**	**η_0 , Pas (25 °C)**	**τ_c, Pa (25 °C)**
**BE57_c1**	0		**-**
**PC66_c1**	0		**-**
**BPh40_c1**	0	-	-
**BPh40_c2**	0	-	-
**BPh40_CLT 1**	8	177	-
**BPh40_CLT 2**	16	4016	76 ± 13
**BPh40_CLT 3**	24	-	654 ± 16
**BPh40_CLT 4**	32	-	2363 ±46

BE57_c1= PC66_c1=BPh40_c1 = 500 mg/mL; BPh40_c2 = 250 mg/mL
